# Induced transcriptional profiling of phenylpropanoid pathway genes increased flavonoid and lignin content in *Arabidopsis* leaves in response to microbial products

**DOI:** 10.1186/1471-2229-14-84

**Published:** 2014-04-01

**Authors:** Mohammad Babar Ali, David H McNear

**Affiliations:** 1 Department of Plant and Soil Sciences, Rhizosphere Science Laboratory, University of Kentucky, Lexington, KY 40546, USA

**Keywords:** *Arabidopsis*, Metabolites, Microbes, Transcriptional profiling, Plant Growth Promoting Rhizobacteria, Soil Builder

## Abstract

**Background:**

The production and use of biologically derived soil additives is one of the fastest growing sectors of the fertilizer industry. These products have been shown to improve crop yields while at the same time reducing fertilizer inputs to and nutrient loss from cropland. The mechanisms driving the changes in primary productivity and soil processes are poorly understood and little is known about changes in secondary productivity associated with the use of microbial products. Here we investigate secondary metabolic responses to a biologically derived soil additive by monitoring changes in the phenlypropanoid (PP) pathway in *Arabidopsis thaliana*.

**Results:**

This study was designed to test the influence of one of these products (Soil Builder™-AF, SB) on secondary metabolism after being applied at different times. One time (TI) application of SB to *Arabidopsis* increased the accumulation of flavonoids compared to multiple (TII) applications of the same products. Fourteen phenolic compounds including flavonols and anothocyanins were identified by mass spectrometry. Kaempferol-3,7-O-bis-α-L-rhamnoside and quercetin 3,7-dirhamnoside, the major compounds, increased 3-fold and 4-fold, respectively compared to control in the TI treatment. The most abundant anthocyanin was cyanidin 3-rhamnoglucoside, which increased 3-fold and 2-fold in TI compared to the control and TII, respectively. Simultaneously, the expression of genes coding for key enzymes in the PP pathway *(phenylalanine ammonia lyase, cinnamate 4-hydroxylase, chalcone synthase, flavonoid-3′-O-hydroxylase, flavonol synthase1* and *dihydroflavonol-4-reductase*) and regulatory genes (production of anthocyanin pigment2, *MYB12, MYB113, MYB114, EGL3,* and *TT8*) were up-regulated in both treatments (TI and TII). Furthermore, application of TI and TII induced expression of the lignin pathway genes (*hydroxyl cinamyl transferase, caffeyl-CoA O-methyl transferase, cinnamyl alcohol dehydrogenase, cinnamyl-CoA reductase*, secondary wall-associated NAC domain protein1, *MYB58* and *MYB63* resulting in higher accumulation of lignin content compared to the control.

**Conclusions:**

These results indicate that the additions of microbially based soil additives have a perceptible influence on phenylpropanoid pathway gene regulation and its production of secondary metabolites. These findings open an avenue of research to investigate the mode of action of microbially-based soil additives which may assist in the sustainable production of food, feed, fuel and fiber.

## Background

One of major challenges in the 21^st^ century is the sustainable production of food, fuel and fiber crops with enhanced functional and nutritive value (e.g. flavonoids and anthocyanins) to meet the demands of an ever-increasing global population [1,2]. To meet this demand requires the development of alternative more sustainable methods for the production and enhancement of value added agricultural commodities in a way that will have minimal impact on the ecosystem. Current agricultural practices are largely based on the use of chemical fertilizers and synthetic pesticides for improved crop growth and yield. However, our dependence and overuse of these fertilizers has resulted in contamination of soil, ground and surface waters. Increasing demand for healthier and more nutrient-dense foods by more health-conscious consumers and an improved environmental awareness has resulted in an increased interest in and a rapid change towards eco-friendly sustainable agricultural farming systems.

One component of this new sustainable production system is the use of microbe-based fertilizers (i.e. biostimulants) containing potential beneficial strains of microorganism and their metabolites many of which have an important role in conditioning the rhizosphere for improved plant growth and nutrient use efficiency [3,4]. Since the 1970’s we have been cognizant of the potential benefits on plant growth of specialized plant growth promoting rhizobacteria (PGPR) [5]. There have been many reports on improvements in plant defense, health and growth, resistance to pathogens, enhanced salt tolerance, and improved nutrient uptake in response to PGPR [6,7] that could have led to the development of novel agricultural applications. In spite of all these advantages, the use of microbial-based products has not been effectively exploited at larger scales to improve plant yields and most certainly not as a means to selectively enhance gene expression and production of beneficial secondary metabolite in crops.

Phenylpropanoids are a large group of polyphenolic compounds that comprise an important class of secondary metabolites such as flavonoids, anthocyanin and lignin in plants [8]. Phenylpropanoids have important functions in flower coloration, pollinator attraction, protection from ultraviolet (UV) light, as signaling molecules between plants and microbes, and as antioxidants [9]. Additionally, when consumed by humans phenylpropanoids offer a myriad of health benefits [10,11]. There have been many studies on the biosynthesis of flavonols and the PP pathway in general via metabolic engineering targeting important agronomic traits such as the production of novel colors and color patterns in ornamentals [8,12]. Many phenylpropanoids act as inducers of plant-microbe symbioses [13], whereas others exhibit broad-spectrum antimicrobial activity and are therefore believed to help plants fight microbial diseases [14]. In addition, several studies have examined how the PP and defense related pathways are regulated by interactions between soil microorganisms and plant roots [15-18]. The genes involved in PP pathway such as *chalcone synthase (CHS), chalcone isomerase (CHI), flavanone 3-hydroxylase (F3H), flavonoid 3’-hydroxylase (F3’H), and flavonol synthase1* (*FLS1*) play important roles in the production of secondary metabolites, while *dihydroflavonol 4-reductase (DFR),* and *leucoanthocyanidin dioxygenase (LDOX)* are involved in the production of colored anthocyanidins (Additional file 1). After production, these products are further modified by glycosylation, acylation, and methylation in a complex process that changes their stability, solubility, or localization, and thereby the biological properties of the conjugated molecules [19].

The transcription factors regulating the expression of these structural genes have been well characterized in plant species including *Arabidopsis*[20]. *MYB11, MYB12,* and *MYB111* encode three functionally redundant *MYB*s regulating the expression of several ‘early’ flavonoid biosynthetic genes [21]. On the other hand, TFs such as *PAP1, PAP2, GL3, TT8* and *TTG1* which are components of the *MYB/bHLH/WDR* (*MBW*) transcriptional complexes mediate the ‘late’ anthocyanin biosynthesis genes [21,22]. The lignin biosynthesis pathway is well-characterized and plays an important role in plant growth, development, increase cell wall integrity, facilitating water transport and providing resistance against pathogen [23-25]. The genes which are involved in lignin biosynthesis are largely regulated at the transcription level and lignin-specific transcription factor *MYB58, MYB63* and *SND1* can induce the biosynthetic pathways for the synthesis of lignin [26,27].

To date there is little research aimed at understanding the influence of microbial products on plant secondary metabolism making it difficult to assess a potential functional relationship(s). Understanding how phenylpropanoid metabolism changes in response to microbes or microbial-based products will help to improve our fundamental understanding of plant biology, and would be useful for the development of natural products aimed at improving crop yield and quality. Preliminary analysis of the product composition shows that it is composed of PGPR related bacteria and use of the product can result in plant growth promotion [28,29]. We hypothesized that microbial-based products, known to improve plant growth and nutrient uptake, can induce the PP pathway and lignin pathway in *Arabidopsis.* Therefore, this study was designed to evaluate how application and the timing of application (single and multiple times) influence the PP pathway in *Arabidopsis*. Quantitative real-time PCR (qRT-PCR) was used in this study for transcriptional profiling of flavonoid and lignin pathway genes, and high performance liquid chromatography (HPLC) and liquid chromatography-electrospray ionization-quadrupole-time of flight-mass spectrometry (LC/ESI-Q-TOFMS/MS) were used to determine flavonoid content. The results show that application of microbial products induced the PP path-way and there was a different response dependent on application timing. In both cases application of the microbial product induced flavonoid and lignin content in *Arabidopsis* leaves compared to an untreated control.

## Results

### Metabolite composition

**Figure 1:**
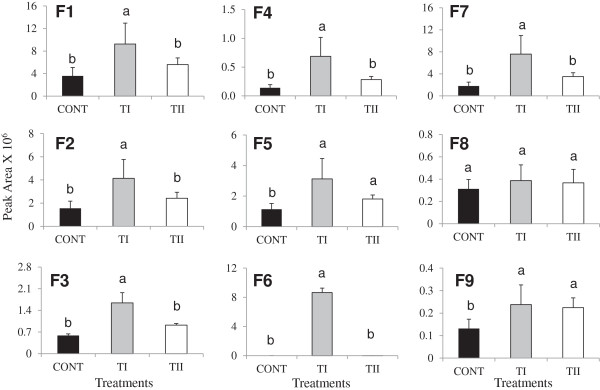
**Profiles of flavonol glycoside detected in *Arabidopsis thaliana *treated once (TI) and multiple times (TII) with SoilBuilder™-AF (SB).** Kaempferol-3,7-O-bis-alpha-L-rhamnoside (F1), kaempferol-3-O-alpha-L-rhamnopyranosyl (1-2)-beta-D-glucopyranoside-7-O-alpha-L-rhamnopyranoside (F2), Kaempferol with rhamnoside (F3), Kaempferol with rhamnoside (F4), Kaempferol with rhamnoside (F5), Kaempferol in hydrolyzed (F6), quercetin 3,7-dirhamnoside (F7), apigenin 7-(2",3"-diacetylglucoside) (F8) and pentamethoxydihydroxyflavone (F9). Bars indicate standard error of three biological replicates at each sampling time-point. Different letters in different bar differ significantly from the control according to Fit Least Squares (FLS) test, *P* ≤ 0.05. CONT (black bar) indicates the untreated plants, TI (shaded) and TII (white) treated with microbial products only once and multiple times, respectively.

**Figure 2:**
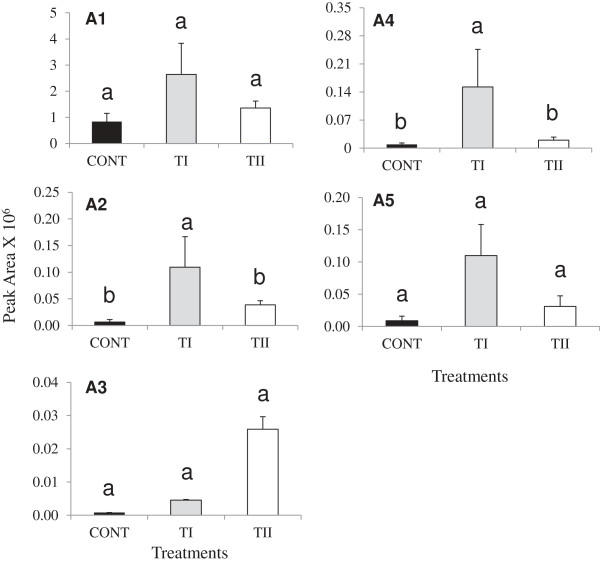
**Profiles of anthocyanidins glycoside detected in *Arabidopsis thaliana *treated once (TI) and multiple times (TII) with SB.** Cyanidin –Rhamnoglucoside (A1), cyanidin 3-(6-malonylglucoside)-7,3’-di-(6-feruloylglucoside) (A2), cyanidin 3-(6"-caffeyl-2"-sinapylsambubioside)-5-(6-malonylglucoside) (A3) and cyanidin 3-(2G-glucosylrutinoside) (A4) and cyanidin 3-(2G-glucosylrutinoside) (A5). Bars indicate standard error of three biological replicates at each sampling time-point. For significant level identification, see Figure 1.

Fourteen flavonoids were identified by HPLC-QTOF-MS/MS analysis in the leaves of *Arabidopsis* (Figures 1 and 2), including nine flavonols: kaempferol-3,7-O-bis-alpha-L-rhamnoside (F1), kaempferol-3-O-alpha-L-rhamnopyranosyl (1-2)-beta-D-glucopyranoside-7-O-alpha-L-rhamnopyranoside (F2), kaempferol with rhamnoside (F3), kaempferol with rhamnoside (F4), kaempferol with rhamnoside (F5), kaempferol in hydrolyzed form (F6), quercetin 3,7-dirhamnoside (F7), apigenin7-(2",3"-diacetylglucoside), (F8), and pentamethoxydihydroxy flavone (F9); as well as five representative anthocyanidins (cyanidin 3-rhamnoglucoside (A1), (cyanidin 3-(6-malonylglucoside)-7,3"-di-(6-feruloylglucoside) (A2), cyanidin 3-(6"-caffeyl-2"-sinapylsambubioside)-5-(6-malonylglucoside) (A3), and two isomers of cyanidin 3-(2G-glucosylrutinoside) (A4 and A5) (Table 1). The majority of *Arabidopsis* flavonoids were found to be anthocyanins, glycosylated kaempferol and rhamnosylated in this study which concurs with previously published findings [30-33].

**Table 1:** **Flavonoids identified in *Arabidopsis thaliana *** **leaf tissue by liquid chromatography-electrospray ionization Q- time of flight - mass spectrometry (LC/ESI- Q-TOF MS/MS) analysis**

**Peak no.**	**Rt (min)**	**ESI-MS *(m/z)***	**Mol formula**	**Compound name**
1 (A4)	7.1294	757.2185	C_33_H_41_O_20_	Anthocyanidin 3-(2G-glucosylrutinoside)
2 (F2)	7.3303	740.2166	C_33_H_40_O_19_	Kaempferol-3-O-alpha-L-rhamnopyranosyl (1-2)-beta-D-glucopyranoside-7-O-alpha-L-rhamnopyranoside
3 (F3)	7.3482	432.1062	C_21_H_20_O_10_	Kaempferol with rhamnosides
4 (A1)	7.4932	595.1674	C_27_H_31_O_15_	Anthocyanidin 3-Rhamnoglucoside
5 (A5)	7.5893	757.2188		Anthocyanidin 3-(2G-glucosylrutinoside)
6 (F5)	7.7591	432.1062	C_21_H_20_O_10_	Kaempferol with rhamnosides
7 (F7)	7.7687	594.1596	C_27_H_30_O_15_	Quercetin 3,7-dirhamnoside
8 (A2)	7.7707	1211.305	C_56_H_59_O_30_	Anthocyanidin 3-(6-malonylglucoside)-7,3′-di-(6-feruloylglucoside)
9 (A3)	7.9512	1197.293	C_55_H_57_O_30_	Anthocyanidin 3-(6"-caffeyl-2"-sinapylsambubioside)-5-(6-malonylglucoside)
10 (F9)	8.1942	362.0622	C_17_H_14_O_9_	Pentamethoxydihroxyflavone
11 (F8)	8.2139	516.1278	C_25_H_24_O_12_	Apigenin 7-(2",3"-diacetylglucoside)
12 (F1)	8.2625	578.1646	C_27_H_30_O_14_	Kaempferol-3,7-O-bis-alpha-L-rhamnoside
13 (F4)	8.2657	432.1067	C_21_H_20_O_10_	Kaempferol with rhamnosides
14 (F6)	11.434	286.048	C_15_H_10_O_6_	Kaempferol

*Rt*, retention time; ESI-MS, electrospray ionization/mass spectrometry; *m/z,* mass/charge.

Significant changes in the biosynthesis of flavonoids occurred that depended on treatment and time of application, except for F8 (Figures 1 and 2). One time application of products (TI) induced the peak area of F1, F2, F3, F4, F5, F6, F7, F8, and F9, compared to control (Figure 1). Similarly, but to a lesser extent, peak area levels of F1, F2, F3, F4, F5, F7, F8 and F9 were increased in the TII treatments compared to control (Figure 1). When compared between TI and TII treatments, TI treatments increased the peak area of F1, F2, F3, F4, F5, F6, F7, F8, and F9 compared to TII. The compound kaempferol, F6, which was detected at Rt11.43 (F6; *m/z*, 286.04) in the hydrolyzed leafy extracts, was induced significantly in the TI treatments compared to control and TII. The peak area of apigenin (a flavones containing compound, F8), did not change with the treatments, but F9 increased significantly in both TI and TII treated plants, while no significant difference was found in the peak area level of F9 between the treatments. The five anthocyanin derivatives (A1-A5) were increased in both TI and TII treated plants compared to control (Figure 2). TI induced the level of A2 and A4 significantly compared to TII and control (Figure 2). Comparing TI and TII treatments, the TI treatment increased the level of A1, A2, A4, and A5) compared to TII. Nevertheless, TII treatment increased the level of A3 compared to TI.

### Expression of flavonoid biosynthesis genes in *Arabidopsis* leaves

**Figure 3:**
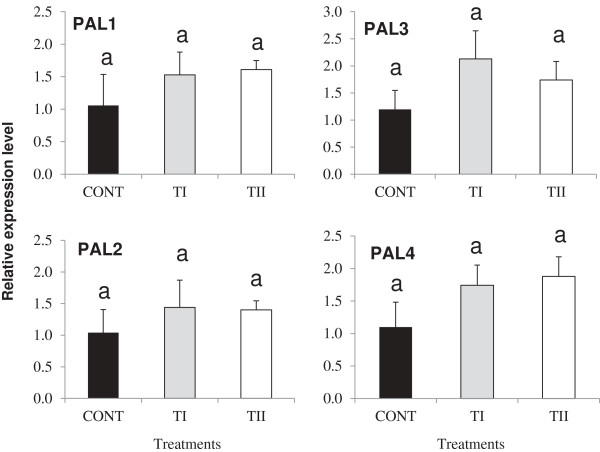
**Relative transcript abundance of phenylalanine ammonia lyase (*PAL*) of flavonoid pathway (*PAL1, PAL2, PAL3 *and *PAL4*) genes known to be involved in flavonoid biosynthesis in *Arabidopsis thaliana *after being treated once (TI) and multiple times (TII) with SB.** Primers used in these studies, products size for the amplified fragments, accession numbers are shown in Additional file 6. Transcript abundance of each gene was normalized by the level of an actin and EF-1α gene. Bars indicate standard error of three biological replicates at each sampling time-point. For significant level identification, see Figure 1.

**Figure 4:**
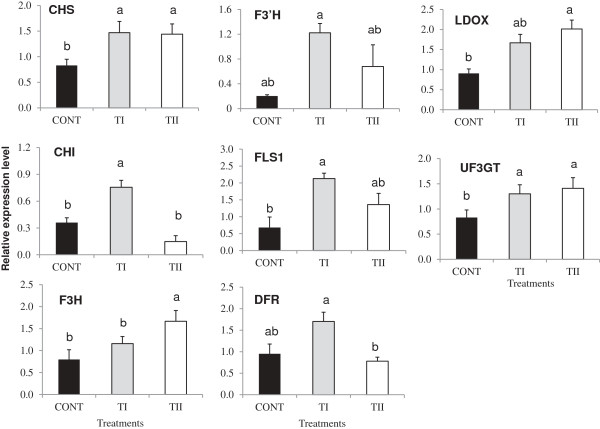
**Relative transcript abundance of flavonoids pathway structural genes (*CHS, CHI, F3H, F3´H, FLS1, UF3GT, DFR *and *LDOX*) known to be involved in flavonoid biosynthesis in *Arabidopsis *treated once (TI) and multiple times (TII) with SB.** Primers used in these studies, products size for the amplified fragments, accession numbers are shown in Additional file 6. Transcript abundance of each gene was normalized by the level of an actin and EF-1α gene. Bars indicate standard error of three biological replicates at each sampling time-point. For significant level identification, see Figure 1.

To understand the influence of microbial product application timing (TI and TII) on the flavonoid pathway, the expression of genes encoding key PP pathway enzymes *PAL1, PAL2, PAL3, PAL4, C4H, CHS, CHI, F3H, F3’H, FLS1, DFR, LDOX,* and *UF3GT* were analyzed in *Arabidopsis* leaves using qPCR (Figures 3 and 4). Both types of treatments (TI and TII) did not induce the expression *PAL1, PAL2, PAL3* and *PAL4* significantly (*P* ≥ 0.05) compared to control (Figure 3). Expression of *CHS, FLS1, LDOX,* and *UF3GT* was induced double in both types of treatments compared to the control, while TI and TII treatments increased expression of *F3’H* 8 and 4 times more compared to control, respectively (Figure 4). Expression of *CHI* increased significantly (*P ≤* 0.05) in TI compared to control and TII treated plants, while *CHI* expression did not change significantly in TII treated plants compared to the control. Expression of *F3H* increased significantly (*P ≤* 0.05) in TII compared to control and TI treated plants, while *F3H* expression did not change significantly in TI treated plants compared to the control. TI treatment increased expression of *DFR* by one fold compared control and no change of *DFR* expression was found in TII treated plants compared to control. Acylation genes (*At1g03495, At1g03940* and *At3g29590*), glycosylation genes (*UGT75C1* and *UGT78D3*), *GST* and *UDP*-rhamnose synthase genes (*RHM1, RHM2,* and *RHM3*) increased in the TI and TII treated plants compared to control in the majority of the cases (see Additional files 2A, B and C).

### Expression pattern of flavonoid pathway regulatory genes in *Arabidopsis* leaves

**Figure 5:**
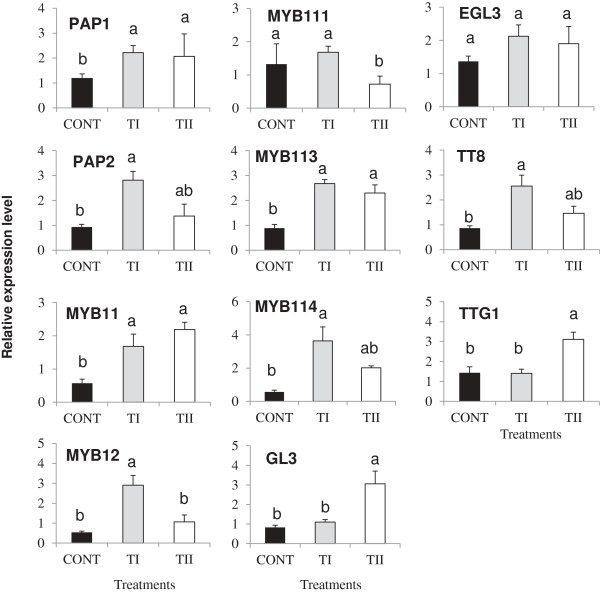
**Relative transcript abundance of transcription factors (*PAP1, PAP2, MYB11, MYB12, MYB111, MYB113, MYB114, GL3, EGL3, TT8 *and *TTG1*) known to direct flavonoids biosynthesis-related gene expression in *Arabidopsis *treated once (TI) and multiple times (TII) with SB.** Primers used in these studies, products size for the amplified fragments, accession numbers are shown in Additional file 6. Transcript abundance of each gene was normalized by the level of an actin and EF-1α gene. Bars indicate standard error of three biological replicates at each sampling time-point. For significant level identification, see Figure 1.

To examine whether the induced expression of flavonoid biosynthetic genes in leaves was accompanied by the expression of regulatory genes, we analyzed the transcript levels of *PAP1, PAP2, MYB11, MYB12, MYB111, MYB113, MYB114, GL3, EGL3, TT8* and *TTG1* in the leaves of *Arabidopsis* treated with TI and TII (Figure 5). Expression of most of the regulatory genes was induced in both TI and TII treated plants compared to control. Expression levels of *PAP1* and *PAP2* were increased in both TI and TII treated plants compared to the control; and even more so for *PAP2* in the TI treated plants, which experienced a 3-fold increase. Expression of *MYB11, MYB12, MYB113* and *MYB114* were increased in both TI and TII treated plants compared to control. Expression of *MYB12* and *MYB114* was induced to the greatest extent in the TI compared to TII treated plants. Expression of *MYB11* and *MYB113* was induced in both TI and TII treated plants compared to control. Conversely, *MYB111* expression in the TII treatment was suppressed, and the TI treatment only slightly up-regulated. The effect of treatment on *GL3* and *TTG1* expression levels was similar with no induction for the TI and control, whereas expression of both genes increased significantly (*P ≤* 0.05) compared to TI and control. Expression of *EGL3* did not change in both TI and TII treated plants compared to control. Strong increase in the expression levels of *TT8* was noted in the TI treated plants compared to control.

### Lignin biosynthesis

**Figure 6:**
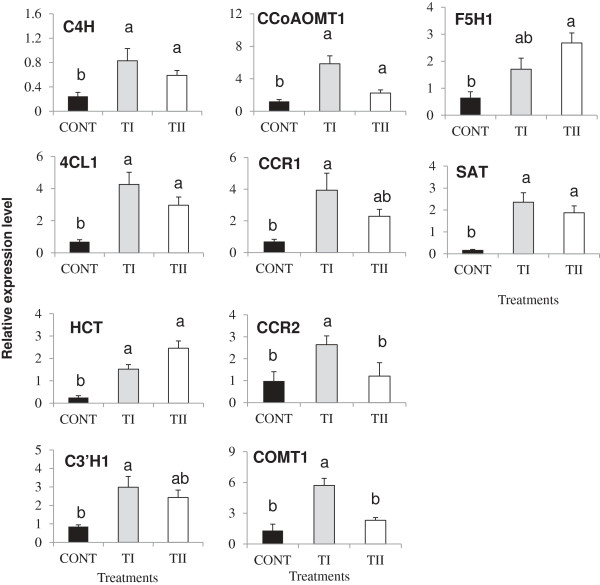
**Relative transcript abundance of structural genes (*C4H, 4CL1, HCT, C3’H1, CCoAOMT1, CCR1, CCR2, COMT1, F5H1, *and *SAT*) known to involved in lignin biosynthesis in *Arabidopsis *treated once (TI) and multiple times (TII) with SB.** Primers used in these studies, products size for the amplified fragments, accession numbers are shown in Additional file 6. Transcript abundance of each gene was normalized by the level of an actin and EF-1α gene. Bars indicate standard error of three biological replicates at each sampling time-point. For significant level identification, see Figure 1.

**Figure 7:**
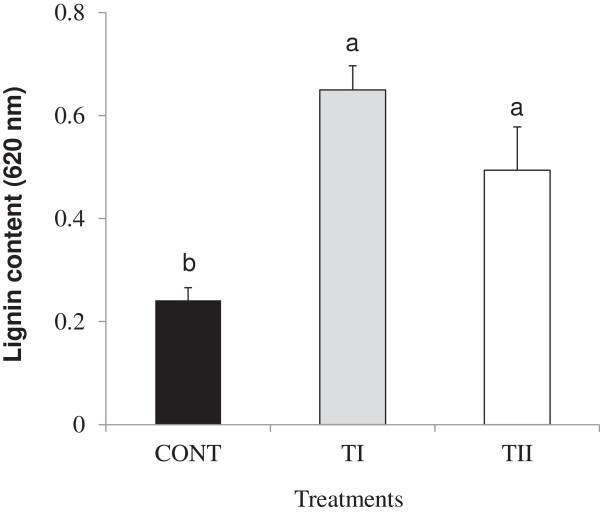
**Influence of microbial products SB treated once (TI) and multiple times (TII) on lignin content in *Arabidopsis thaliana*.** Bars indicate standard error of three biological replicates at each sampling time-point. For significant level identification, see Figure 1.

To further understand the application of microbial treatments (TI and TII), we analyzed the expression of all the genes (20) involved in the lignifications pathway (Figures 3, 6, 7 and Additional file 3). The accumulation of transcripts for *C4H, 4CL1*, *C3’H1, CCoAOMT1, CCR1, CCR2, COMT1, CAD1, CAD3, CAD4, CAD5, CAD7, CAD8* and *SAT* were induced significantly (*P ≤* 0.05) in TI treated plants compared to control. Expression levels of *HCT*, *F5H1,* and *SAT* were increased significantly (*P ≤* 0.05) in TII treated plants compared to control. No significant (*P* ≥ 0.05) difference of expression levels of *C3’H1, CCR1, CCR2,* and *COMT1* were observed between control and TII treated plants. We found that expression levels of *CCR2, CAD1, CAD5, CAD7,* and *CAD8* were increased significantly (*P ≤* 0.05) in TI compared to TII treated plants. Expression of *LAC4*, *LAC17,* and their regulatory genes (*SND1, MYB58,* and *MYB63*) increased significantly (*P ≤* 0.05) in both TI and TII treated plants compared to control (Additional file 4), and also with significant (*P ≤* 0.05) expression levels for the TI compared to the TII treated plants. Significant (*P ≤* 0.05) accumulation of lignin was noted in the TI and TII treated plants compared to control.

## Discussion

### Expression of flavonoid pathway genes and metabolite composition

Our study provides evidence that application of microbial products (TI and TII) to *Arabidopsis* plants increases the accumulation of flavonoids, and that TI resulted in greater accumulation of metabolites than TII treated plants. Such enhancement was accompanied by an increased expression of most of the genes in the flavonoid biosynthetic pathway. This was particularly prominent in TI treated plants, but there were also elevated expression levels in TII treated plants. Synthesis of the derivatives of kaempferol, quercetin, and anthocyanins depend greatly on dihydrokaempferol. Meanwhile, *F3’H* and *FLS1* are crucial to channeling the precursor’s dihydroquercetin/dihydrokaempferol, forming quercetin or kaempferol branches. The up-regulation of *F3’H* and *FLS1* in TI compared with TII treated plants are consistent with an increase in the amounts of most of flavonol glycosides in *Arabidopsis* leaves (Figure 1). However, in TII treated plants, there was a significant decrease in the amounts of most of flavonol glycosides compared to TI, which is also in accordance with the down-regulation of *F3’H* and *FLS1* observed in TII treated plants. Such difference may be due to the variability of specific substrate, which can make the biosynthetic pathway different, thus producing different products. Therefore, the increase of kaempferol-containing flavonols is primarily associated with the accumulation of F1, which is 2-fold higher as compared to F2, F3, F4, F5, F7, and F9 in TI treated plants.

LC-MSMS analyses detected a strongly increased level of a compound (F6) in acid hydrolyzed samples corresponding to molecular mass (*m/z,* 286.0) of aglycone kaempferol. Consistent with the induced expression of *DFR, LDOX* and *UF3GT* in both TI and TII treated plants, we observed significant increases in the amount of anthocyanins (A1, A2, A3, A4 and A5) in the leaves of *Arabidopsis* as compared to the control (Figure 2). The effect was most prominent for A1, A2, A4 and A5 in the TI treated plants wherewith higher accumulation was observed compared to those plants receiving the TII treatment. In contrast, plants treated with TII showed higher accumulation of A3 compared to control. The presence of caffeyl, ferulyl, sinapyl and malonyl groups in the accumulated anthocyanins is parallel with the induced expression of anthocyanin acyltransferases, and *SAT* in the present study [34]. The accumulation of these compounds could provide some level of increased defense against biotic and abiotic stresses. For example, the presence of the acylated flavonol glycosides in the leaf hairs of *Quercus ilex* increased the plant’s protection against the damage of UV-B radiation [35]. In another example, rutin showed significant antifungal activity against the fungi *Cylindrocar pondestructans*, *Phytophthora megasperma*, and *Verticillium dahlia* attacking olive trees (*Olea europaea*). Rutin is, therefore, believed to play a major role in plant defense [36]. The formation of a large number of glycoslated kaepmferol derivatives and only a small amount of glycosylated anthocyanidins in our study are corroborated by earlier reports [37,38].

Several *GT*s that are involved in the glycosylation processes and induced expression of *UGT75C1* and *UGT78D3*[30,38] are consistent with the expression of *FLS1*, *F3’H,* and glycosylated flavonol compound biosynthesis in TI and TII treated plants. Rhamnosylation is a major glycosylation process of flavonols and the genes responsible for biosynthesis of rhmanose sugar, and is vital in supplying *UDP*-rhamnose for flavonol biosynthesis. Enhanced expression of *RHM1, RHM2* and *RHM3* in the TI and TII treated plants suggests their possible roles in the formation of Rha residue and rhamnosylation of flavonoids. Our LC-MSMS data also suggest that most flavonol accumulation in the microbial treated plants is in the rhamnosylated form. Induced expression of rhamnosylated genes was reported in *Arabidopsis*[31], confirming previous results that suggest its involvement in rhamnosylation of flavonols in TI and TII treated plants. The induced expression of *RHM1, RHM2,* and *RHM3* in our study may be due to either competition of substrate availability, or to changes in the flow of flux in the different branches of the PP pathway, a phenomenon which has been reported in *Arabidopsis*[39,40]. These results showed significant variation in flavonoid accumulation, indicating that the accumulation of flavonoid may potentially be manipulated by altering the application timing of the microbial based products.

### Transcriptional regulation of flavonoid pathway genes

Transcriptional regulation of flavonoid biosynthesis structural genes are controlled by regulatory genes, which provide an additional level of control. Several *MYB/bHLH/WD*-repeat (*MBW*) family genes have been implicated in the regulation of flavonoid biosynthesis in *Arabidopsis*. Among them, *PAP1* is a master regulator of flavonoid/anthocyanin biosynthesis pathway [41]. Our results suggest that induced expression of *PAP1* and its close homolog, *PAP2,* are strongly induced in TI and TII treated plants, regulating the flavonoid biosynthesis. Interestingly, *PAP2* expression was increased in TI treated plants as compared to *PAP1*. Thus, it is reasonable to suggest that *PAP2* expression was stable enough to control flavonoid biosynthesis.

There are several other genes encoding *MYB* and *bHLH* transcription factors (TF) that are known to be involved in regulating flavonoid biosynthesis in *Arabidopsis* leaves. Therefore, the expression of *MYB11, MYB12, MYB111, MYB113, MYB114, GL3, EGL3, TT8* and *TTG1* was analyzed. *TT8* interacts with *PAP1/PAP2*[42], and the up-regulation of *PAP1/PAP2* and *TT8* genes appears to be required for the activation of anthocyanin structural genes for anthocyanin production in TI treated plants. The closely related *MYB11, MYB12*, and *MYB111* TFs are flavonol-specific regulators, and effect flavonol accumulation in different parts of the *Arabidopsis* seedlings by regulating several genes of flavonoid biosynthesis, such as *CHS, CHI, F3’H,* and *FLS1*[21]. Consistent with the up-regulation of *PAP1* and *PAP2*, induced expression of *MYB11, MYB12, MYB113* and *MYB114* genes resulted in an increase in the content of individual flavonol biosynthesis in the TI compared TII treated plants. However, induced expression of *PAP1, MYB11* and *MYB113* gene coincided with reduced expression of *PAP2, MYB12, MYB111* and *MYB114* in the TII treated plants, suggesting a balance among *MYB* gene members in controlling the flavonoid biosynthesis in our study. Variable regulation of the TFs and regulatory genes in the TII treated plants likely led to the lower amounts of flavonols and anothcyanin and higher amounts of F8, F9 and A3 observed in these plants.

Interestingly, a significant increase in the amounts of A3 was noted in the TII as compared to TI treated plants that may have been due to the induced expression of regulatory genes *GL3* and *TTG1,* and anthocyanin biosynthetic genes *LDOX* and *UF3GT*. Previous studies have indicated that the mutant of *ttg1* disrupted the expression of late anthocyanin biosynthetic genes such as *DFR* and *LDOX*, whereas the expression of ‘early’ anthocyanin biosynthetic genes (*CHS, CHI,* and *F3H*) is not effected in the same mutant [22,43,44]. Our results show that genes involved in the biosynthesis of flavonoids are expressed differently in TI and TII treated plants, which explains why some of the flavonoids are produced in much higher amounts as compared to the control. This clearly indicates some correlation between the biosynthesis of these closely related flavonoids in response to plants receiving the microbe-based soil additive. However, further work is required to understand whether these changes are due to the microbes or metabolites in the product, or the interaction of the two, and if they are acting directly on the plant or indirectly by mediating the interaction of the plant with the surrounding soil.

### Expression of transcription factors during lignin accumulation

A branch of the PP pathway is responsible for the synthesis of lignins by the coordinated transcription of lignin pathway genes (*C4H, 4CL1, C3’H1, CCoAOMT1, CCR1, CCR2, COMT1, CAD1, CAD3, CAD5, CAD7, CAD8* and *SAT*) [45-49]. TI strongly induced the expression of *C4H, 4CL1, HCT, C3’H1, CCoAOMT1, CCR1, CCR2, COMT1, CAD1, CAD3, CAD5, CAD7, CAD8* and *SAT*, whereas TII induction of lignin biosynthesis genes is relatively low, with the exception of *HCT* and *F5H1,* whose expression levels were higher in the TII compared to TI treated plants (Figure 6 and Additional file 3). *C4H, 4CL1,* and *HCT* have been shown to be involved in lignification [50-52]; furthermore, the increase in the expression levels of *C4H, 4CL1,* and *HCT* in the TI and TII treated plant could be linked to the lignification process. *CCoAOMT1* and *COMT1* expression was induced in TI and TII treated plants. *CCoAOMT1* encodes an enzyme involved in monolignol biosynthesis by catalyzing the methylation of caffeyl-CoA ester. Moreover, in TI treated plants, the up-regulation *CCoAOMT1* and *COMT1* were observed more than those of TII treated plants. Overexpression of *PAP1* (a positive regulator of anthocyanin biosynthesis) in *Arabidopsis* showed increased amounts of guaiacyl and syringyl monomers that were associated with increased lignin accumulation [24]. In *Vitis vinifera*, *VvMYB5a*, which regulates anthocyanin and proanthocyanidin biosynthesis in grapevine, also affects lignin biosynthesis. Overexpression of *VvMYB5a* in tobacco down regulated *CCoAOMT1* gene expression, leading to reduced lignification in anther walls and delayed dehiscence [53]. It was also observed that *C4H* and *COMT1* genes are regulated by a lignin-specific *MYB* transcription factor *MYB58* in *Arabidopsis*[54,55].

Of the two *CCR* isogenes, *CCR1* showed higher overall expression levels than *CCR2* in both TI and TII treated plants, but only plants form TI had greater *CCR1* expression compared to control. Up-regulation of *CCR* expression has been associated with an increase in lignin formation in *Arabidopsis*[56]. Nine *CAD* genes have been reported in *Arabidopsis*[48]. The results showed that six members of the *CAD* family genes (*CAD1, CAD3, CAD4, CAD5, CAD7,* and *CAD8*) accumulated at varying levels, with *CAD1, CAD3, CAD7* and *CAD8* expression being higher in the TI treated plants compared to TII treated plants (Additional file 3). *CAD4* and *CAD5* have been shown to play a major role in lignifications [57]. We observed that *CAD4* and *CAD5* expression was induced to the same degree in both TI and TII treated plants compared to control. A role of *CAD1* in lignification has been shown in young stems, flowers, and siliques of *Arabidopsis*[58]. *CAD3* expression was detected in secondary growth in hypocotyls of *A. thaliana*[59]. A several fold increase in the levels of expression of *CAD7* and *CAD8* was noted in the TI treated plants compared to TII treated plants; however, their expression was increased in the TII treated plants compared to control plants. The higher induced expression levels of *CAD7* compared to *CAD8* was also observed in plantlets and flowers of *Arabidopsis*[58]. Thus, different members of the *CCR* and *CAD* family genes appear to be induced differently in lignin biosynthesis in plants treated with microbe-derived products. The induced expression of these genes in our study suggested that they might contribute to the biosynthesis of lignin. *At2g23000,* encoding sinapoylglucose:anthocyanin sinapoyltransferase (*SAT*), plays a role in sinapoylmalate synthesis [60] an increase in the expression levels of *SAT* in the TI and TII treated plant was also noted.

Both *CCR* and *CAD* are critical for lignin biosynthesis, transferred into the cell wall, and polymerized by laccases [61,62]. The up-regulation of laccases (*LAC4* and *LAC17*) is accompanied by the up-regulation of several TFs in both TI and TII treated plants responsible for controlling lignin biosynthesis (Additional file 4). It was shown that *LAC4* is expressed in vascular bundles and interfascicular fibers; and, that *LAC17* contributes in the interfascicular fibers lignifications [63]. Secondary wall associated NAC domain protein1 (*SND1*), a key transcriptional activator regulating the developmental program of secondary wall biosynthesis [64], was induced in TI and TII treated plants compared to control (Additional file 4). *MYB58* and *MYB63* have been suggested to be specific activators for lignin biosynthesis [55]. The induced expression of these genes in both TI and TII treated plants (Additional file 4) suggested that they are actively involved in the lignifications process.

## Conclusions

This study shows that microbial products applied to the soil of growing plants support our hypothesis and results in induction of the PP pathway and increased metabolite biosynthesis. The one time application of microbial products (TI) produced more metabolites than multiple applications (TII). The higher metabolite biosynthesis in TI compared to TII could be explained by the fact that both TI and TII contained microbial products, while TII applied few times more compared to TI may have indirectly inhibit the metabolite biosynthesis or diverted the metabolites to other metabolic pathway. However, overall flavonoid accumulation was higher in the treated plants, regardless of timing, as compared to the control. Such differences in the flavonols and anthocyanin accumulations between TI and TII treated plants can be explained by the differential transcript accumulation of structural and regulatory genes in leaves of *Arabidopsis*. This is one of the first studies to show that microbial products play an important role in activating the PP pathway in leaves of *Arabidopsis*. These results suggest that the mechanism(s) responsible for the enhancement of metabolites could be related to the microorganisms or metabolites in the product, or an interaction of both. Innovative approaches are needed such as pyro-sequencing for the identification of specific microbial groups, and metabolomics analysis for the identification of possible bioactive metabolites within the product to evaluate those responsible for activation of the transcriptional cascade observed in this study.

## Methods

### Source of microbial preparation

Soil Builder™-AF, SB (Advanced Microbial Solutions, Pilot point, TX, USA), a biochemical fertilizer catalyst developed specifically for the agriculture industry, contains bacteria products derived from a bioreactor system consisting of a large and diverse microbial community. The microbial community composition of SB has been assessed using 16S rRNA based gene analysis and is generally composed of species of bacillus, actimomyces and proteobacteria. Previous works also reported that SB consists of bacillus species, actinomycetes, cyanobacteria, algae, protozoa, and microbial by-products [65] including microbial metabolites produced during anaerobic fermentation of a microbial community [66]. Basic chemical composition of the product was determined by the University of Kentucky Soil Testing Laboratory following standard protocols (Additional file 5).

### Growth conditions and treatment procedure

Seeds of *Arabidopsis thaliana* ecotype Columbia-0 were sterilized and sown on solid 0.7% agar plates containing 1× Murashige and Skoog medium (pH 5.7). Plates were incubated in darkness at 4°C for 2-3 days and were transferred to a growth chamber at 22°C with a 16-h light/8-h dark cycle at a photosynthetic photon flux density (PPFD) of 100 μmol m^-2^ s^-1^, and 65-70% relative humidity and grown for two weeks. After two weeks seventy of the seedlings were transferred to six inch pots containing fertilizer (PRO-MIX_®_ BX, Quakertown, PA, USA), arranged in randomized complete block design in the growth chamber and allowed to acclimate for 7-10 days. Plants were treated following the manufacturers recommended application rate of 100 ml of 6× concentrated 16 ml/L SB. For TI, individual plants were treated with the products only on 1^st^ day, and in parallel with same solutions for 1^st^ day and every 3^rd^ day for TII. Control plants were treated with same water in every 3^rd^ day. Each set of treatment have 18-20 plants including control. On day 14, from each set 5-6 plants were divided randomly to collect representative of biological replicates (R1, R2 and R3). Leaves were collected on day 14 (control and treated), weighed, and were immediately frozen in liquid nitrogen and stored at -80°C until RNA extraction.

### RNA extraction, cDNA synthesis and Quantitative real-time PCR (qPCR)

Total RNA was extracted from three biological replicates using TRI-ZOL method following the manufacturer instructions. cDNA synthesis and qPCR analysis was done according to the method of [67]. Transcript levels in *Arabidopsis* were measured in triplicates of each biological replicate by qPCR, using SYBR Green (Applied Biosystem) in the Applied Biosystem StepOnePlus™ Real-Time PCR Systems following the manufacturer’s manual. The relative mRNA levels were determined by normalizing the PCR threshold cycle number of each gene with that of the *EF-1*α and ACTIN as reference genes, according to GeneEx software ( http://www.multid.se/order/bioeps.php;) and the data were the average of three replicates. Sequences of primers used in this study were retrieved from literature and used for amplifying gene-specific sequences (Additional file 6[24,25,27,31,48,68-77].

### Identification and quantification **of flavonoids and anthocyanins** by LC-ESI-QTOF-MS/MS method

Metabolite profiling was performed as described by Lea et al. (2007) with some modifications. Approximately 100 mg frozen leaves from three biological were homogenized under liquid nitrogen and transferred to a 2 mL Eppendorf tube, to which was added 1 mL of methanol. The phenolics were extracted for 18 h at room temperature in darkness, and centrifuged at 14,000 rpm for 15 min. The extract was filtered through a sterile Syr filter (25 mm, 0.20 μm) through sterile syringe (MicroLiter Analytical Supplies, Inc). Acid hydrolysis was performed by adding 30 -μl of 3 N HCL into the sample, and then heated for an hr at 70°C in water bath. The accuracy of machine was tested in the extract of samples before running analyses, and we injected a mix of flavonoids to make sure flavonoids were working in LC-MSMS method that included the following: Apigenin-7-glucoside, liquiritigenin, geninstein, quercetin, apigenin, and epicatechin. Profiling of metabolites (flavonoids and anthocyanins) in leaf extracts was performed using an LC system with pump model 1200 with 6530A (Agilent Technologies, CA, USA) quadrupole time of flight (Q-TOF) mass spectrometer equipped with an Agilent Jet Stream electrospray ionization (ESI) ion source. The ESI source used a separate nebulizer for the continuous introduction of reference mass compounds: 121.050873, 922.009798 (positive ion mode). Five microliters of sample extract was separated using an Acquity BEH Shield RP-18 analytical column (1.7 μm 2.1×150 mm, Waters Corporation, Milford, CT) maintained at 40°C. The mobile phase of solvent A consists of water/formic acid (99.9:0.1, v/v) and (B) acetonitrile/formic acid (99.9:0.1, v/v) with a solvent ratio of A:B of 95:5. The following gradient for binary pump 1 was used with a total analysis time of 21 min and a flow rate of 0.25 mL/min: 5% to 25% mobile phase B over 2 min then to 25% to 65% mobile phase B for 2.0 to 10.5 min, then to 99% mobile phase B for 10.5 to 12.5 min, then held at 99% mobile phase for 12.5 to 14 min followed by to 5% B from 14 to 15.5 min and then held at 5% 15.5 to 17 min.

The analytical conditions of mass spectrometry are as follows: range, start (100 amu), stop (1,700 amu), and scan time (4.0 s); heating gas temperature, 350°C; gas flow (l/min), 8.0; nebulizing gas, 35 psi; Sheath gas temp, 350; Sheath gas flow 11.0; VCap 3000; nozzle voltage (V) 1000. The fragmentor voltage was 120 V and skimmer1 65 VandoctopoleRFPeak 750 and collision energies (20 eV) were optimized for each compound. To confirm the identity of the flavonoids, MS/MS (*m/z*) fragmentation patterns were compared with those of previously published reports [41] and confirmed by accurate mass Q-TOF analyses. In the absence of authentic standards, the flavonoids were quantified by peak area. MSMS spectra were compared with LC ESI-Q-TOF- MS/MS spectra of known compounds from the ReSpect data base containing all flavonoids MS/MS spectra (published by Prof Kazuki Saito, JP) and Metlin (the Agilent MS/MS spectral library). Results are reported as the mean of the three replicates.

### Lignin measurement

Lignin was measured quantitatively according to [78]. Leaves were homogenized in liquid N_2_. The powdered was transferred to a tube and suspended in 1.5 ml of methanol, and vigorously stirred for 1 h. The homogenate was centrifuged for 15 min at 14,000 rpm, the pellet was washed with 1.5 ml of the following solvents by vortexing for 15 min: (i) methanol twice; (ii) 1 M NaCl; (iii) 1% SDS; (iv) water twice; (v) CHCl_3_/CH_3_OH (1:1); and (vi) tert-butyl methyl ether. The final pellet was freeze-dried overnight, and 10 mg was taken for the following assay. The powder was treated with 1 ml of 2 M HCl and 0.2 ml of thioglycolic acid for 4 h at 95°C. The mixture was cooled down and centrifuged for 10 min. The pellet was washed with water three times, and dissolved in 1 ml of 0.5 M NaOH and the suspension was shaken vigorously overnight at 4°C to extract the lignin thioglycolic acid derivatives (LTGAs), and centrifuged for 10 min. The supernatant was collected in a fresh tube, and the pellet was mixed with 0.5 ml of 0.5 M NaOH for 1 h. Following centrifugation, the supernatant was combined with the previous one, and acidified with 0.3 M concentrated HCl to precipitate LTGAs at 4°C for 4 h. The mixture was centrifuged, and the pellet was kept overnight at 4°C. The LTGA pellet was dissolved in 1.5 ml of 0.M NaOH and the absorption was measured at 280 nm on a spectrophotometer. Results are reported as the mean of the three replicates.

### Statistical analysis

Statistical analyses of quantitative qRT-PCR data were performed by the GenEx software (MultiD analysis) and JPM9 (SAS Institute Inc, Cary, NC, USA). Bars indicate standard error of three biological replicates at each sampling time-point. Different letters in different bar differ significantly from the control according to Fit Least Squares (FLS) test, *P* ≤ 0.05. CONT (black bar) indicates the untreated plants, TI (shaded) and TII (white) treated with microbial products only once and multiple times, respectively.

## Abbreviations

*4CL1*: 4-coumarate:CoA ligase1; *C3′H1*: *p*-coumarate 3-hydroxylase1; *C4H*: Cinnamate 4-hydroxylase; *CAD*: Cinnamyl alcohol dehydrogenase; *CCoAOMT*: Caffeoyl-CoA *O*-methyltransferase; *COMT1*: Caffeic acid *O*-methyltransferase1; *HCT*: HydroxycinnamoylCoA: shikimate/quinatehydroxycinnamyltransferase; *CCoAOMT1*: Caffeyl CoA 3-*O*-methyltransferase 1; *CCR1*: Cinnamoyl CoA reductase1; *CAD*: Cinnamyl alcohol dehydrogenase; *PAL*: Phenylalanine ammonia-lyase; *CHS*: Chalcone synthase; *CHI*: Chalcone isomerase; 0*UF3GT*: UDP-glucose:flavonoid-3-O-glucosyltransferase; *F3H*: Flavanone 3-hydroxylase; *F3′H*: Flavonoid-3′-O-hydroxylase; *DFR*: Dihydroflavonol-4-reductase; *LDOX*: Leucoanthocyanidin dioxygenase; *UDP-GST*: UDP-glucoronosyl/UDP-glucosyltransferase; *GST*: Glutathione S-transferase; *FLS1*: Flavonol synthase1; *PAP1 & 2*: Production of anthocyanin pigment1 & 2; *EGL3*: Enhancer of glabra3; *GL3*: Glabrous 3; *3GlcT*: Flavonoid 3-*O*-glucosyltransferase; *3RhaT*: Flavonol 3-*O*-rhamnosyltransferase; *7GlcT*: Flavonol 7-*O*-glucosyltransferase; *5GlcT*: Anthocyanin 5-*O*-glucosyltransferase; PGPR: Plant growth-promoting rhizobacteria; SB: Soil Builder™-AF.

## Competing interests

The authors declare that they have no competing interests.

## Authors’ contributions

M.B.A. conceived, designed the experiment, collected the sample, performed total RNA extraction, RNA quality, qRT-PCR, analyzed data, sequencing the qRT-PCR products, metabolite analyses, prepared figures and tables and wrote the initial manuscript and finalized the written manuscript. DJM conceived, designed the experiment and contributed to manuscript editing. Both authors read and approved the final manuscript.

## Supplementary Material

Additional file 1 **The flavonoid biosynthesis pathway that leads to the production of anthocyanidins and flavonols in *Arabidopsis*; several transcription factors (*MYB, bHLH* and WD-40) are indicated along the branches that are likely involved in the transcriptional regulation of the structural genes.** The transcription factors are indicated in black box, structural genes in red color and intermediate products in blue color. Click here for file

Additional file 2 **Relative transcript abundance of acetyltranseferase genes (*At1g03495, At1g03940,* and *At3g29590* ) known to be involved in the acylation (A) UDP-glucosyltransferase (*At4g14090* and *At5g17030* ) and *GST*s (*At1g02920)* genes known to be involved in the glycosylation (B), and rhamnose synthesis genes (*RHM1, RHM2* and *RHM3*) involved in the rhamnosylation (C) of flavonoids treated once (TI) and multiple times (TII) with SB in *Arabidopsis thaliana.*** Primers used in these studies, products size for the amplified fragments, accession numbers are shown in Additional file 6. Transcript abundance of each gene was normalized by the level of an actin and EF-1α gene. Bars indicate standard error of three biological replicates at each sampling time-point. For significant level identification, see Figure 1. Click here for file

Additional file 3 **Relative transcript abundance of cinnamyl alcohol dehydrogenase family genes (*CAD1, CAD3, CAD4, CAD5, CAD7 *and *CAD8*) known to be involved in lignin biosynthesis treated once (TI) and multiple times (TII) with SB in *Arabidopsis thaliana.*** Primers used in these studies, products size for the amplified fragments, accession numbers are shown in Additional file 6. Transcript abundance of each gene was normalized by the level of an actin and EF-1α gene. Bars indicate standard error of three biological replicates at each sampling time-point. For significant level identification, see Figure 1. Click here for file

Additional file 4 **Relative transcript abundance of laccase (*LAC4 *and *LAC17*) genes known to be involved in lignin biosynthesis (A) and transcription factors (*SND1*, *MYB58* and *MYB63*) (B) are known to regulate lignin biosynthesis in *Arabidopsis* treated once (TI) and multiple times (TII) with SB in *Arabidopsis thaliana.*** Primers used in these studies, products size for the amplified fragments, accession numbers are shown in Additional file 6. Transcript abundance of each gene was normalized by the level of an actin and EF-1α gene. Bars indicate standard error of three biological replicates at each sampling time-point. For significant level identification, see Figure 1. Click here for file

Additional file 5 Basic chemical properties of Soil Builder used in the present study. Click here for file

Additional file 6 **Primers used for qRT-PCR analysis and expected size for the amplified fragments.** The accession number from public database is given below. Click here for file
