# Supplementary figures and images for: Induced transcriptional profiling of phenylpropanoid pathway genes increased flavonoid and lignin content in Arabidopsis leaves in response to microbial products
Source: BMC Plant Biol. 2014 Apr 1;14:84. doi: 10.1186/1471-2229-14-84 (PMC4021374; doi:10.1186/1471-2229-14-84)

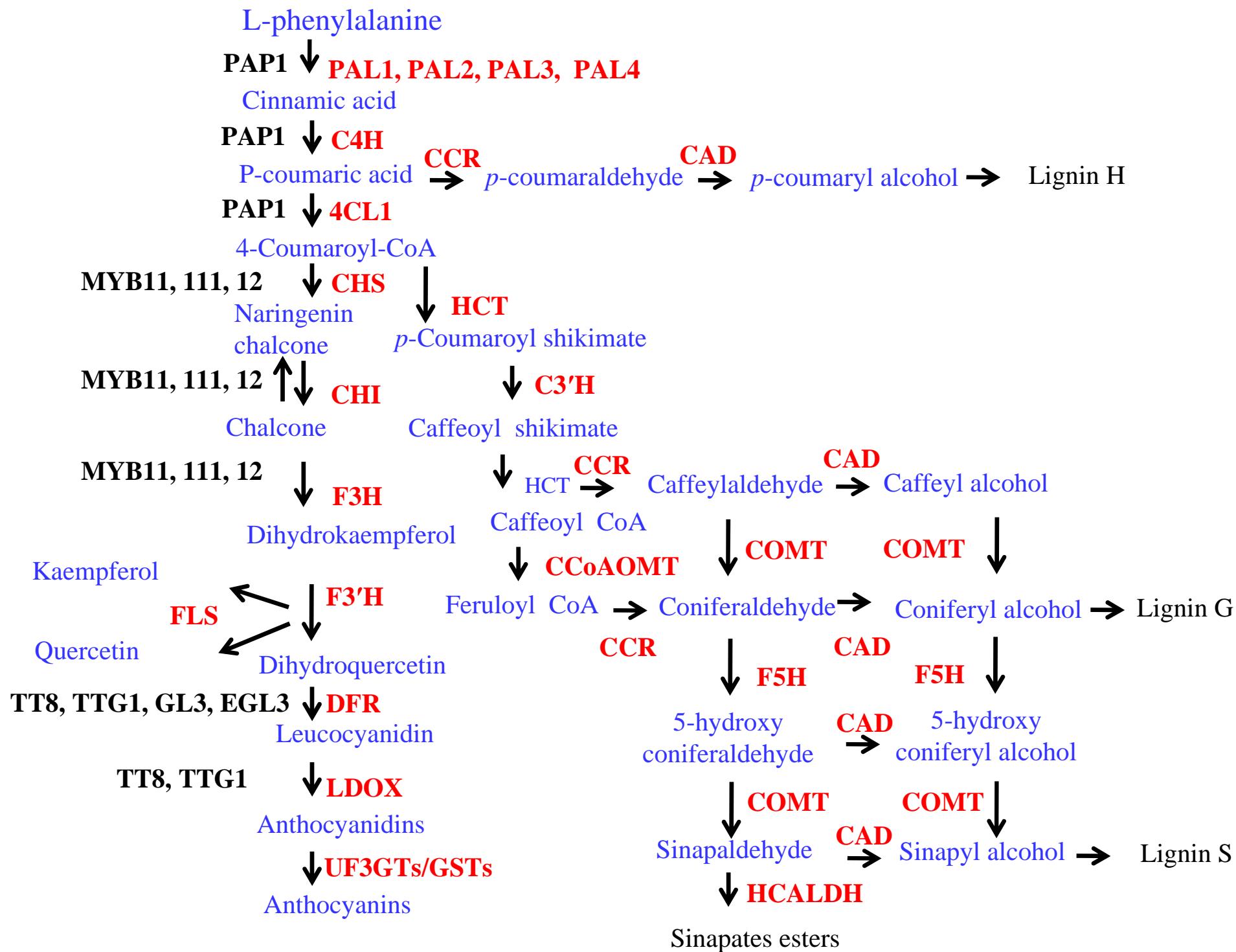

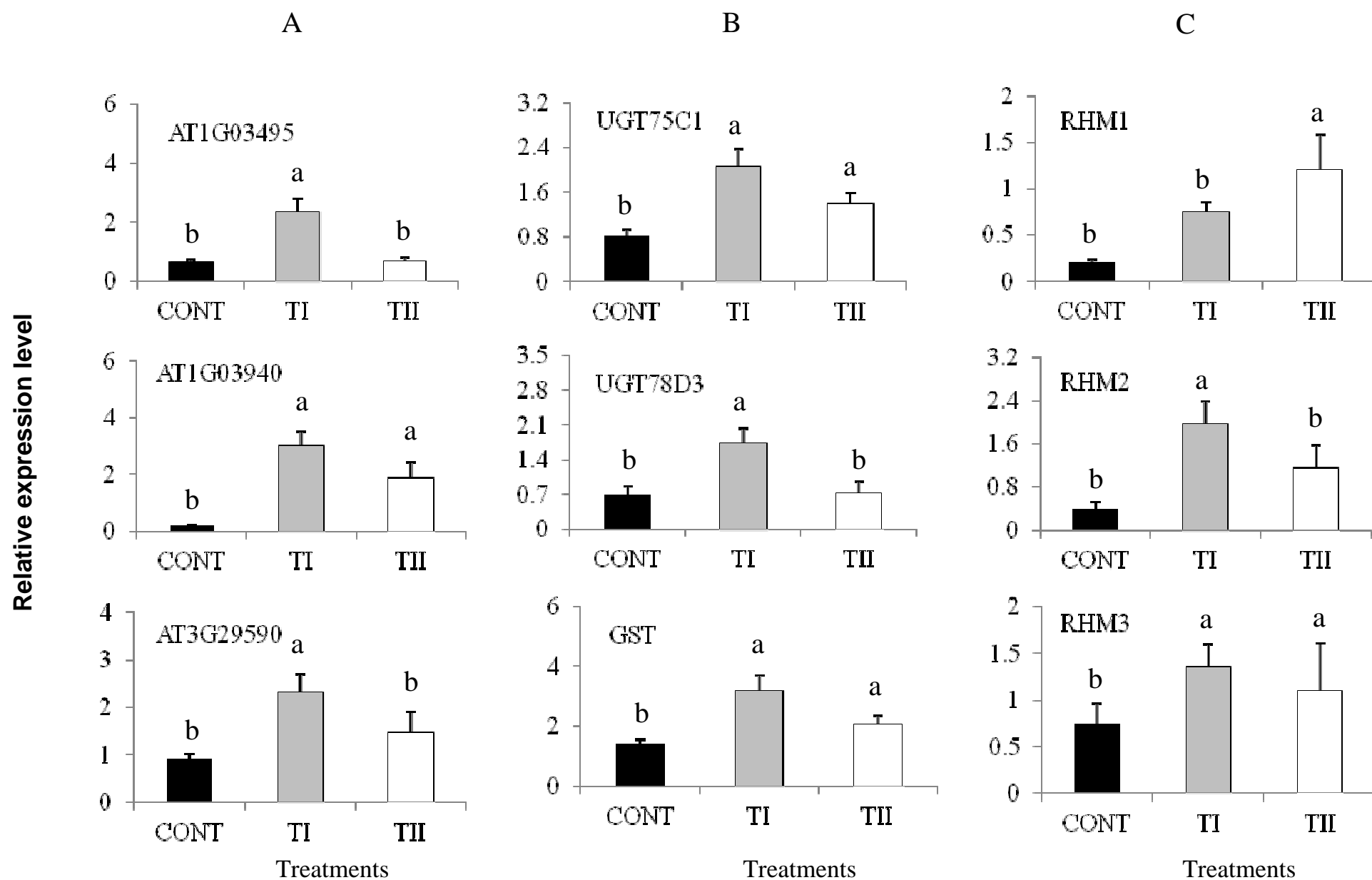

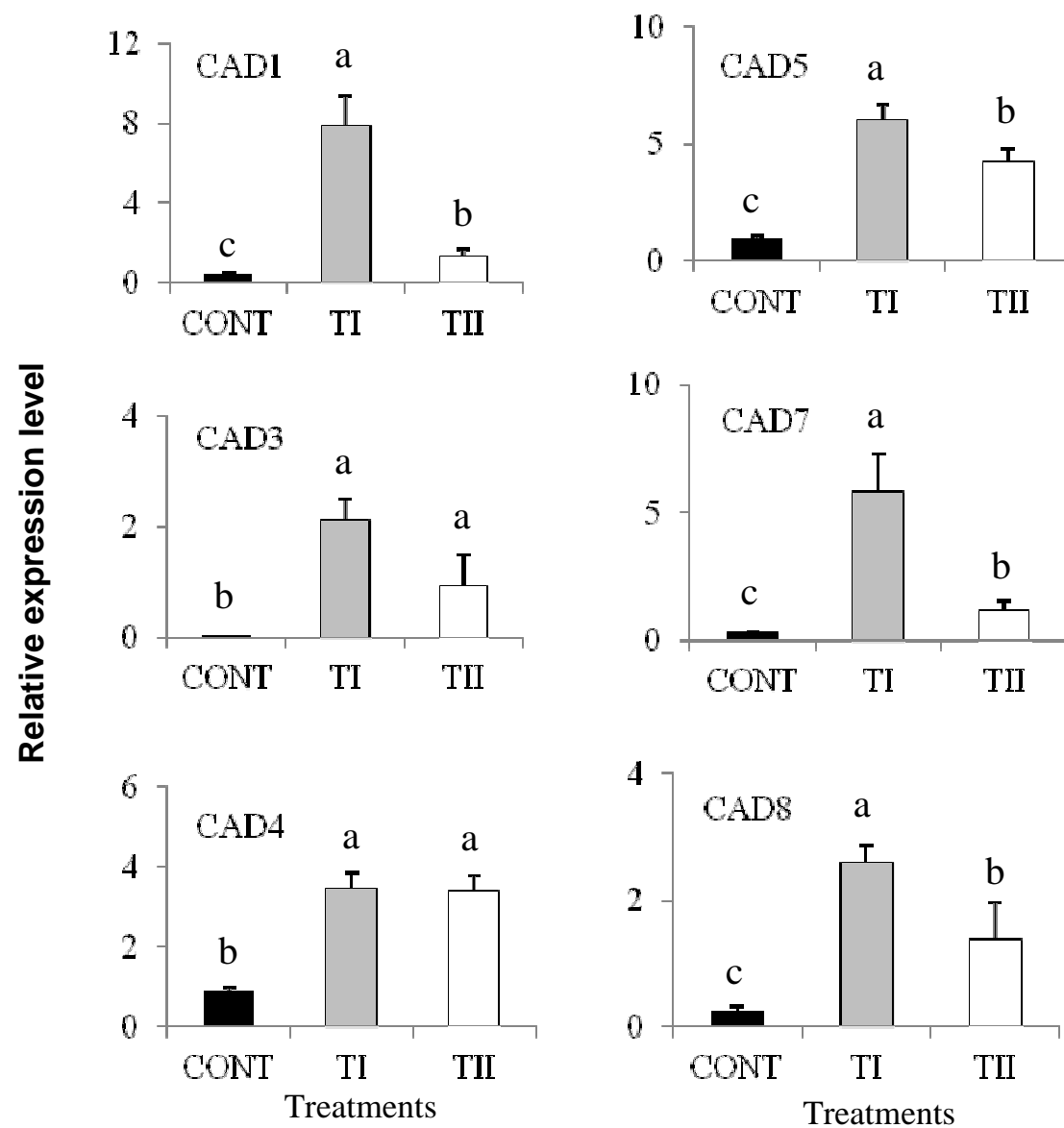

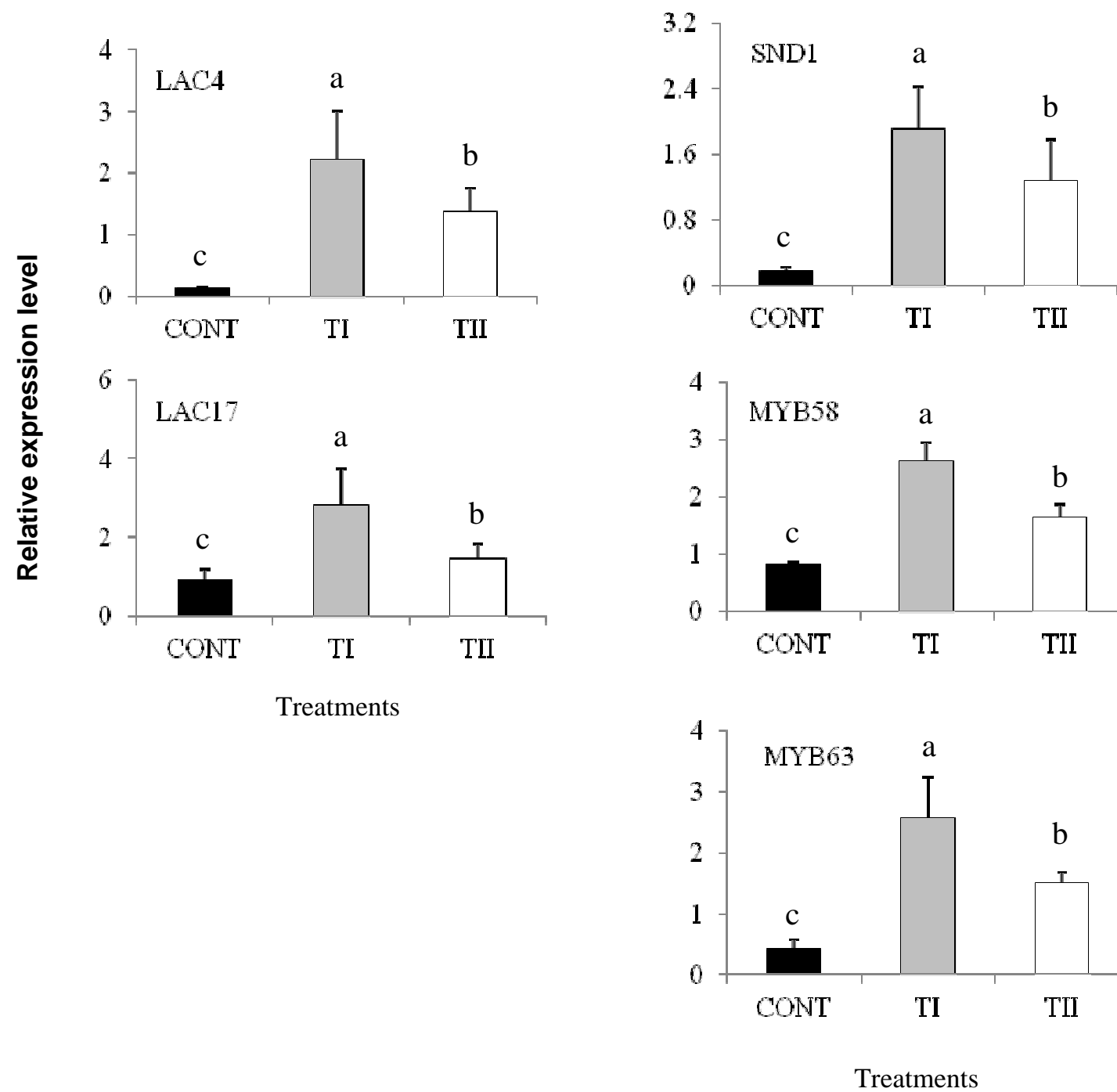

Supplement: Additional file 2 — Relative transcript abundance of acetyltranseferase genes (At1g03495, At1g03940, and At3g29590 ) known to be involved in the acylation (A) UDP-glucosyltransferase (At4g14090 and At5g17030 ) and GSTs (At1g02920) genes known to be involved in the glycosylation (B), and rhamnose synthesis genes (RHM1, RHM2 and RHM3) involved in the rhamnosylation (C) of flavonoids treated once (TI) and multiple times (TII) with SB in Arabidopsis thaliana. Primers used in these studies, products size for the amplified fragments, accession numbers are shown in Additional file 6. Transcript abundance of each gene was normalized by the level of an actin and EF-1α gene. Bars indicate standard error of three biological replicates at each sampling time-point. For significant level identification, see Figure 1. [file 1471-2229-14-84-S2.pdf]

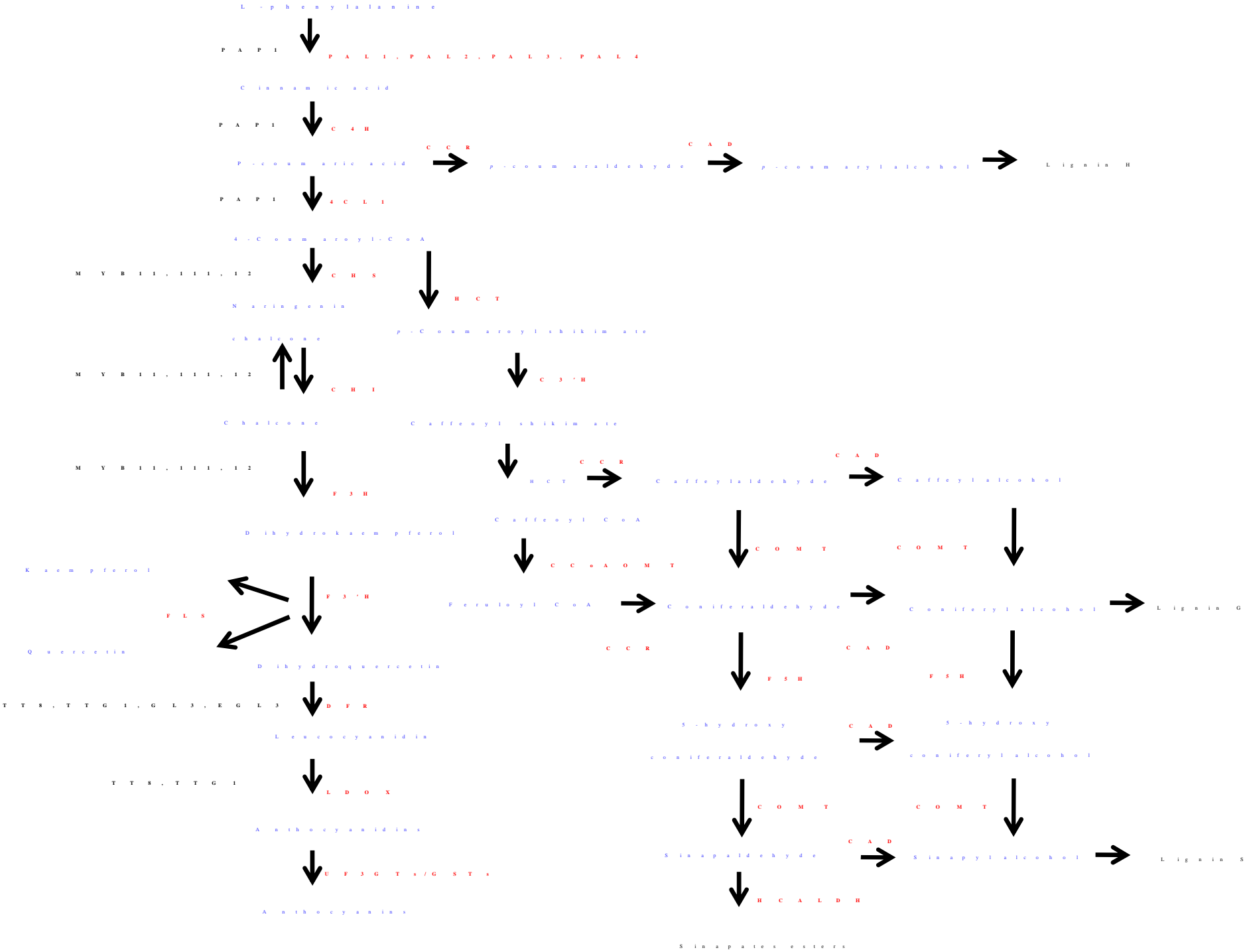

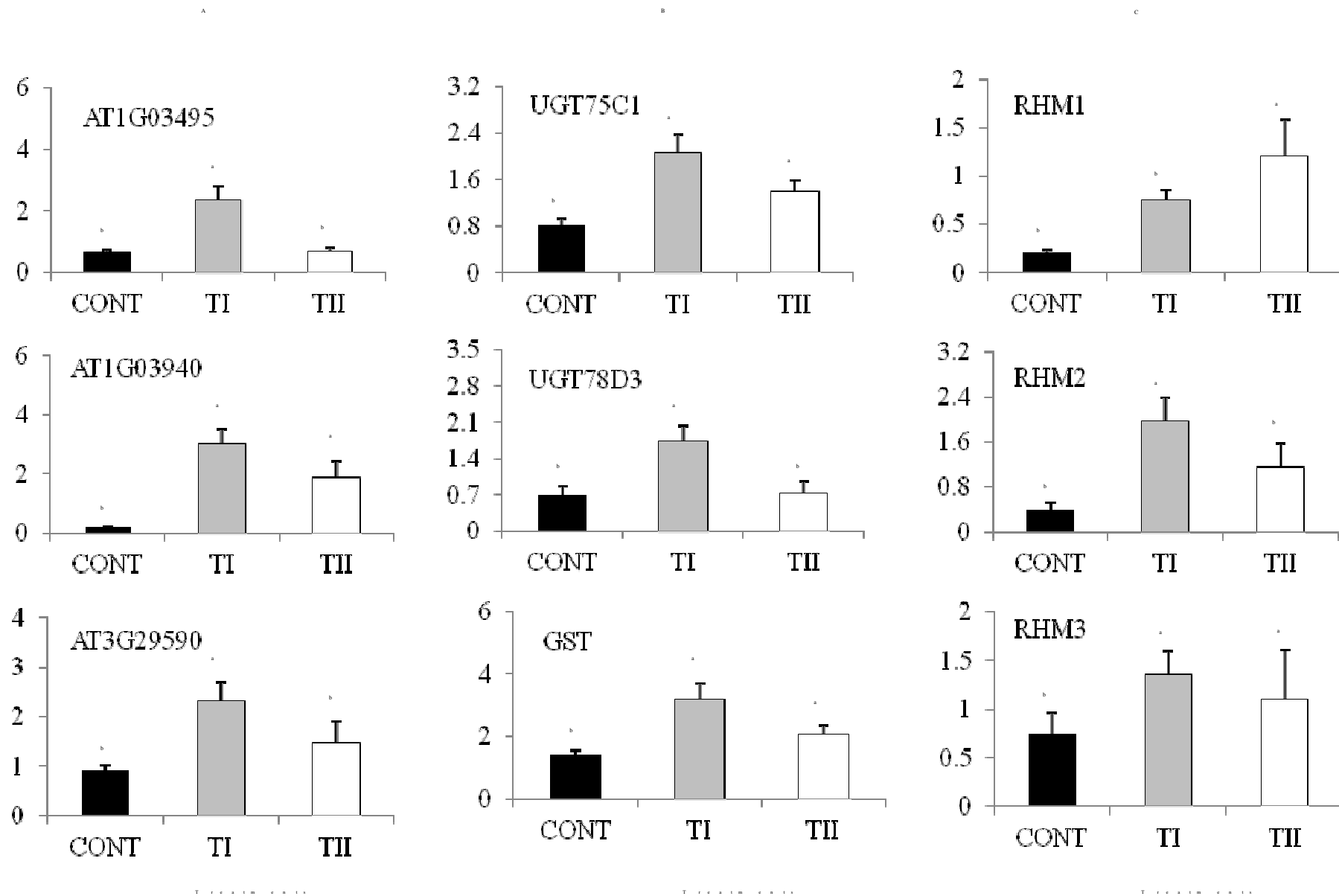

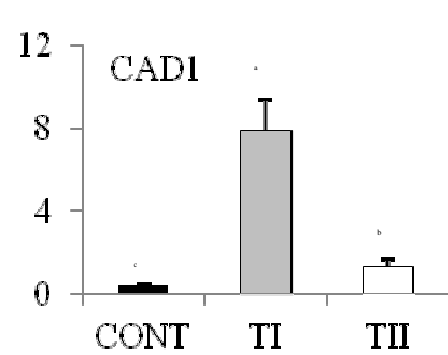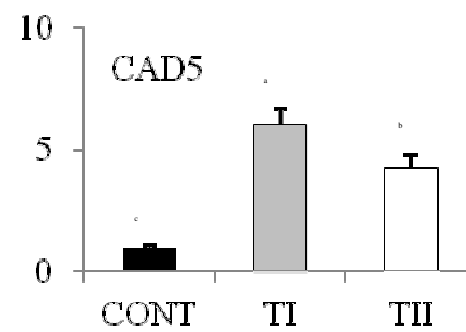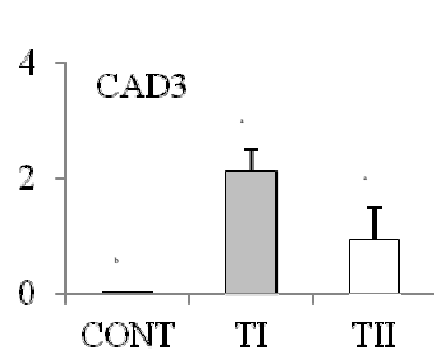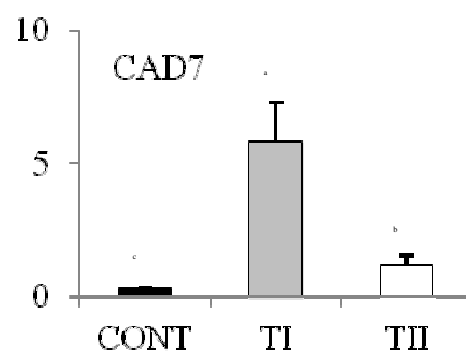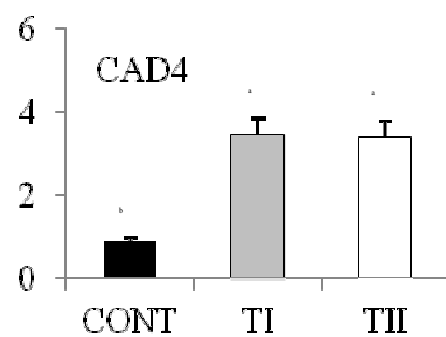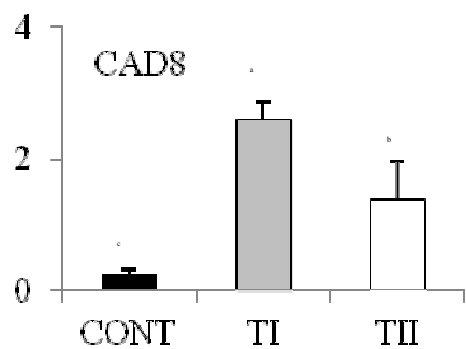

TREATMENT

TREATMENT

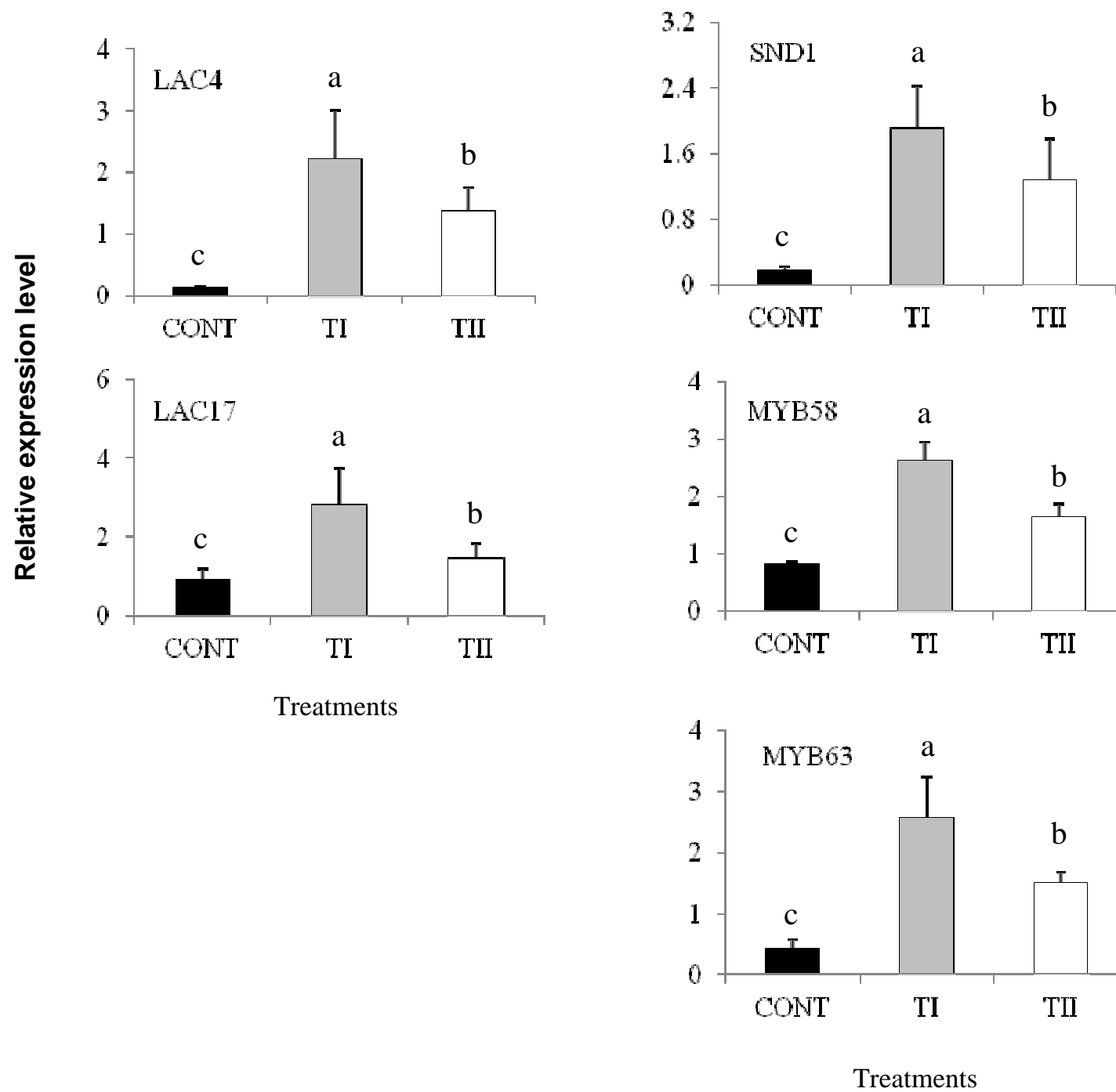

Supplement: Additional file 3 — Relative transcript abundance of cinnamyl alcohol dehydrogenase family genes (CAD1, CAD3, CAD4, CAD5, CAD7 and CAD8) known to be involved in lignin biosynthesis treated once (TI) and multiple times (TII) with SB in Arabidopsis thaliana. Primers used in these studies, products size for the amplified fragments, accession numbers are shown in Additional file 6. Transcript abundance of each gene was normalized by the level of an actin and EF-1α gene. Bars indicate standard error of three biological replicates at each sampling time-point. For significant level identification, see Figure 1. [file 1471-2229-14-84-S3.pdf]

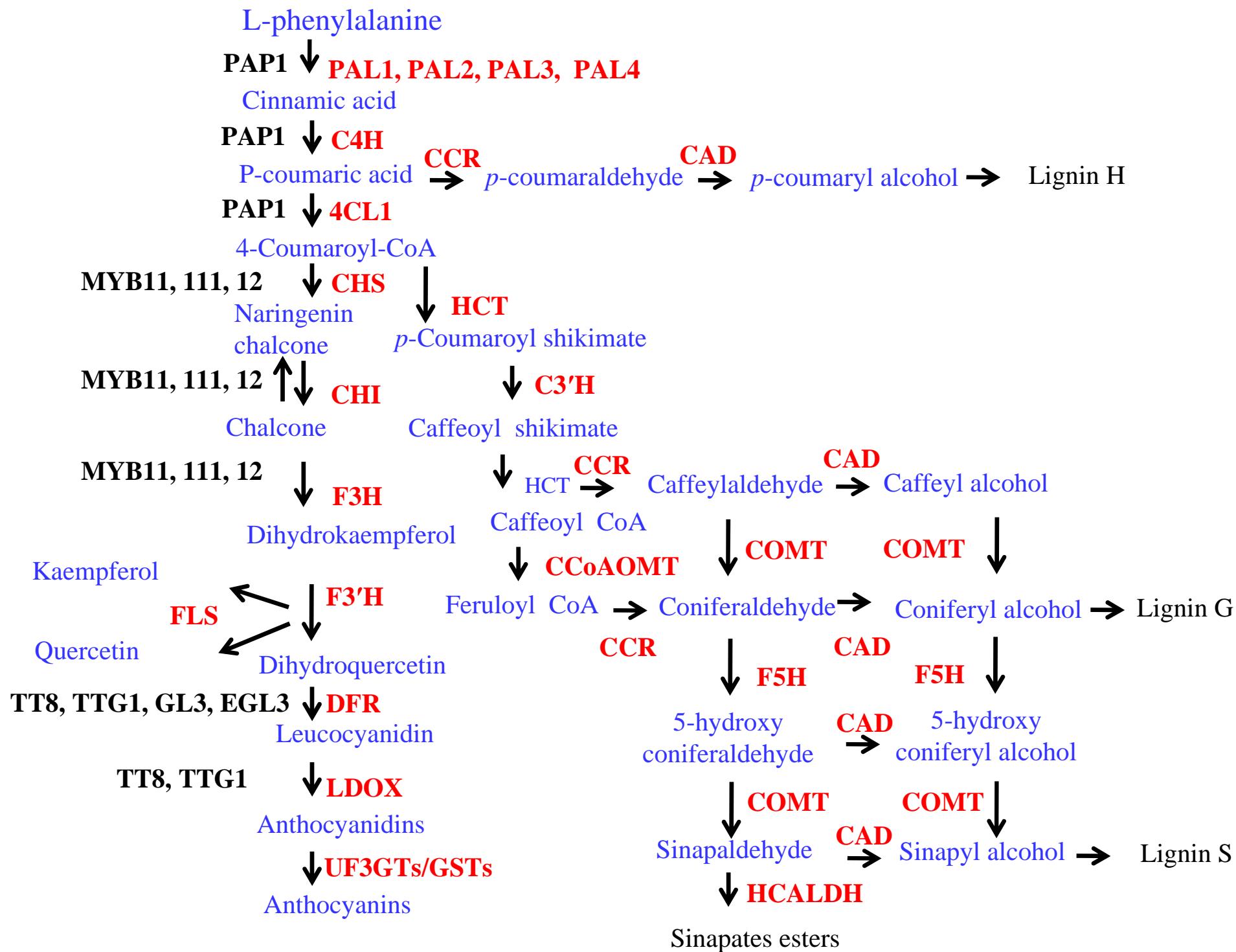

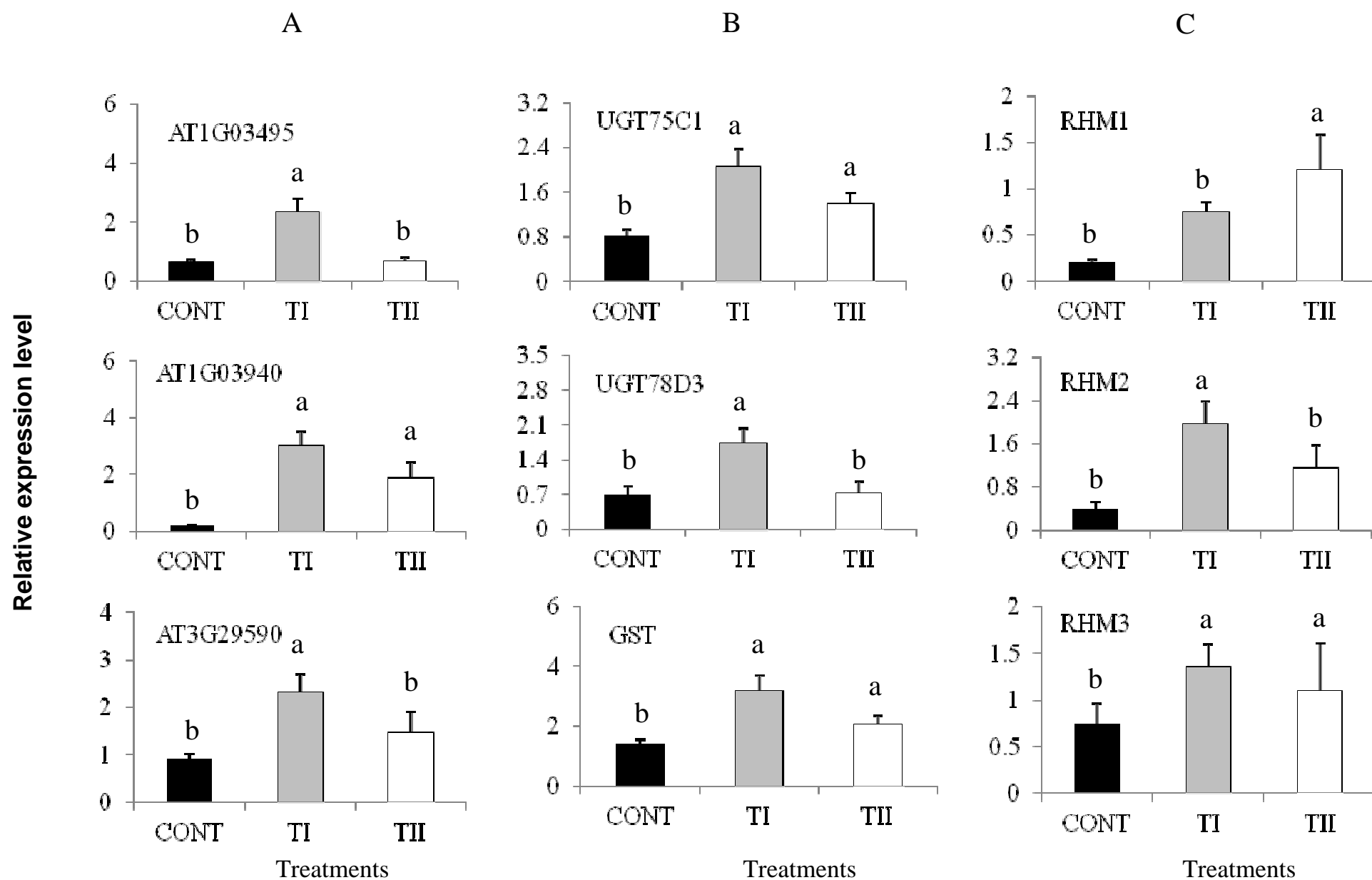

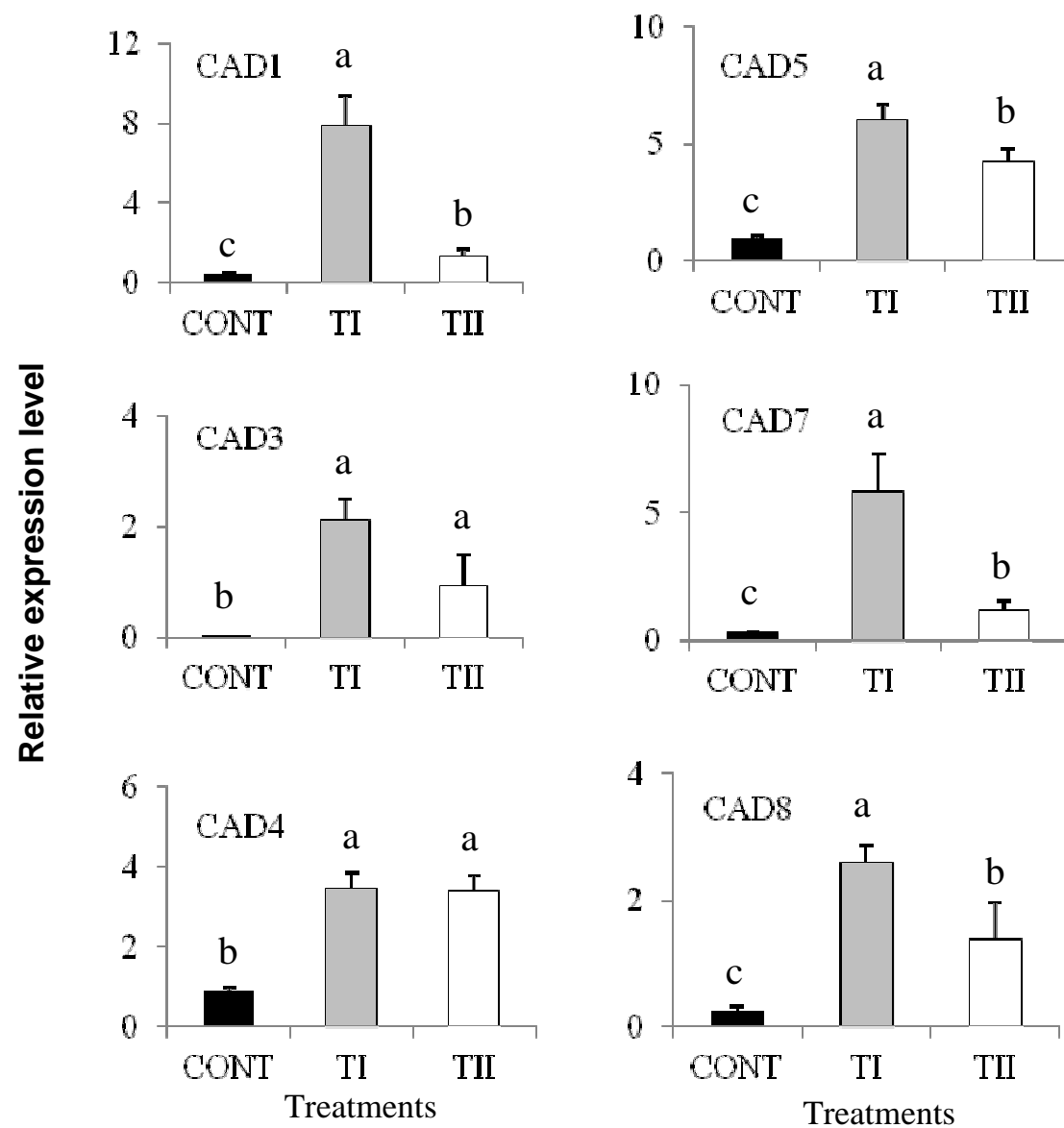

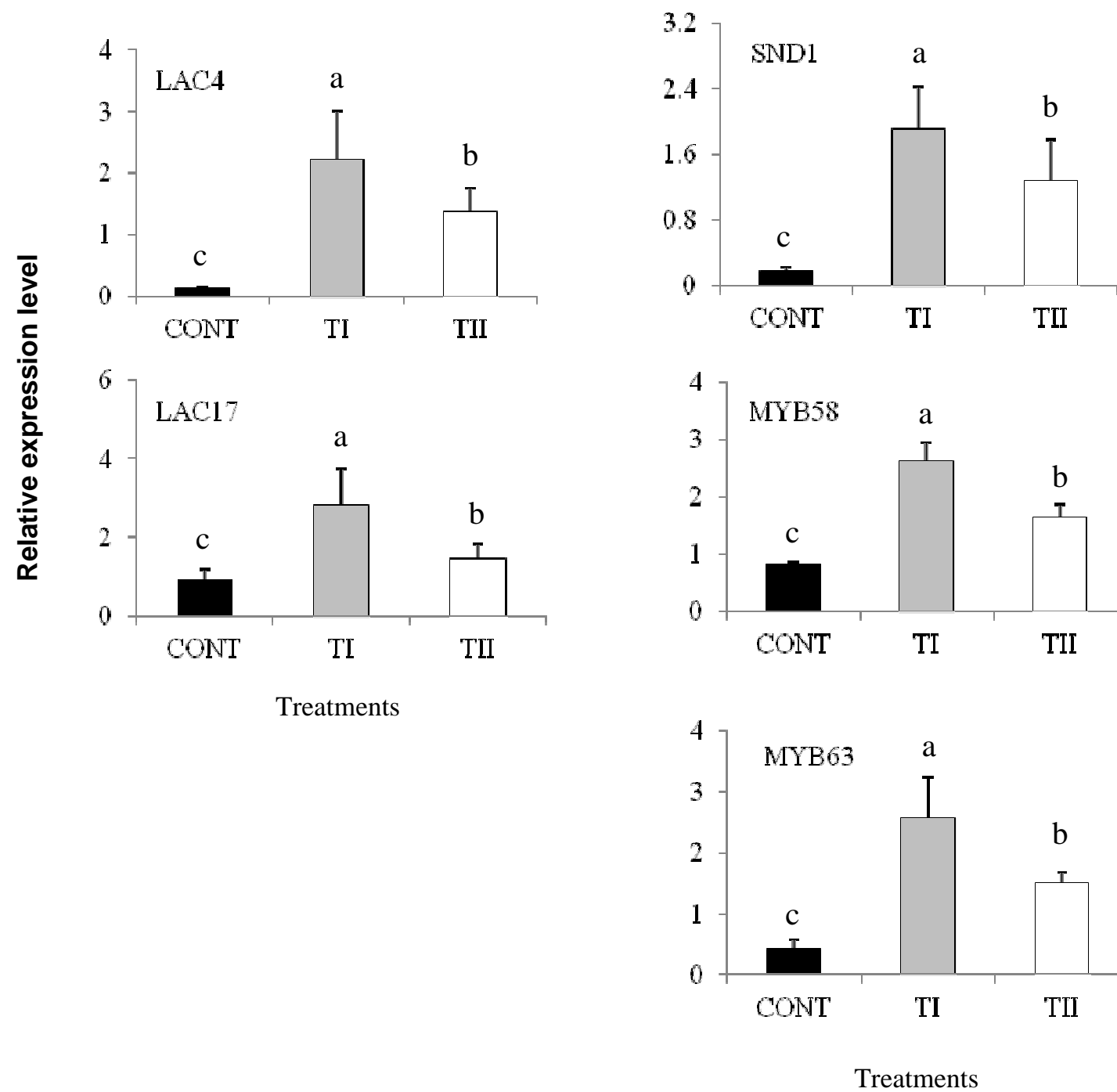

Supplement: Additional file 4 — Relative transcript abundance of laccase (LAC4 and LAC17) genes known to be involved in lignin biosynthesis (A) and transcription factors (SND1, MYB58 and MYB63) (B) are known to regulate lignin biosynthesis in Arabidopsis treated once (TI) and multiple times (TII) with SB in Arabidopsis thaliana. Primers used in these studies, products size for the amplified fragments, accession numbers are shown in Additional file 6. Transcript abundance of each gene was normalized by the level of an actin and EF-1α gene. Bars indicate standard error of three biological replicates at each sampling time-point. For significant level identification, see Figure 1. [file 1471-2229-14-84-S4.pdf]
