# Supplementary material for: Induced transcriptional profiling of phenylpropanoid pathway genes increased flavonoid and lignin content in Arabidopsis leaves in response to microbial products
Source: BMC Plant Biol. 2014 Apr 1;14:84. doi: 10.1186/1471-2229-14-84 (PMC4021374; doi:10.1186/1471-2229-14-84)
Supplement: Additional file 5 — Basic chemical properties of Soil Builder used in the present study. [file 1471-2229-14-84-S5.doc]

Additional file

The following materials are available in the online version of this article.

Additional file 5. Basic chemical properties of Soil Builder used in the present study.

| Properties | Soil Builder |
| --- | --- |
| pH | 8.5 |
| Conductivity (mmho/cm X 0.1=S/m) | 0.88 |
| Alkalinity* | 562 |
| Nitrate-N* | 41 |
| Boron* | 0.3 |
| Phosphorus* | 11.5 |
| Potassium* | 33.3 |
| Calcium* | 23.1 |
| Magnesium* | 8.2 |
| Zinc* | 0.5 |
| Copper* | 1.4 |
| Iron* | 0.3 |
| Manganese* | 0.2 |
| Sodium* | 186.8 |

*Units are in ppm
