# Supplementary material for: Induced transcriptional profiling of phenylpropanoid pathway genes increased flavonoid and lignin content in Arabidopsis leaves in response to microbial products
Source: BMC Plant Biol. 2014 Apr 1;14:84. doi: 10.1186/1471-2229-14-84 (PMC4021374; doi:10.1186/1471-2229-14-84)
Supplement: Additional file 6 — Primers used for qRT-PCR analysis and expected size for the amplified fragments. The accession number from public database is given below. [file 1471-2229-14-84-S6.doc]

Additional file 6**.** Primers used for quantitative real-time PCR and expected size for the amplified fragments. The accession number from public database is given below.

| Gene name | Accession number | Sequence of forward (F) and reverse (R) primers | Expected size (bp) | Reported by |
| --- | --- | --- | --- | --- |
| *PAL1* | At2g37040 | F: GTGTCGCACTTCAGAAGGAA  R: GGCTTGTTTCTTTCGTGCTT | 72 | Huang et al. (2010) |
| *PAL2* | At3g53260 | F: GTGCTACTTCTCACCGGAGA  R: TATTCCGGCGTTCAAAAATC | 77 | Huang et al. (2010) |
| *PAL3* | At5g04230 | F: CAACCAAACGCAACAGCA  R: CTCCAGGTGGCTCCCTTTTA | 78 | Huang et al. (2010) |
| *PAL4* | At3g10340 | F: GGTGCACTTCAAAATGAGCT  R: CAACGTGTGTGACGTGTCC | 81 | Huang et al. (2010) |
| *C4H* | At2g30490 | F: TGAGTTTGGATCCAGAACGAG  R: CGTCATGATTCTTCTCATCTTCCT | 115 | **In this study** |
| *CHS* | At5g13930 | F: CGCATCACCAACAGTGAACAC  R: TCCTCCGTCAGATGCATGTG | 101 | Kleindt et al. (2010) |
| *CHI* | At3g55120 | F: CCGGTTCATCGATCCTCTTC  R: ATCCCGGTTTCAGGGATACTATC | 88 | Kleindt et al. (2010) |
| *F3H* | At3g51240 | F: CAGATCGTTGAGGCTTGTGAGA  R: ACGAGTCATATCCGCCACTAAGT | 88 | Mehrtens et al. (2005) |
| *F3’H* | At5g07990 | F: GCTCTCGCCGGAGTATTCAA  R: CCAGCGACGCCTTGTAAATC | 74 | Mehrtens et al. (2005) |
| *DFR* | At5g42800 | F: AACGGATGTGACGGTGTTTT  R: TCCATTCACTGTCGGCTTTA | 93 | Kleindt et al. (2010) |
| *LDOX* | AT4g22880 | F: CGATGAAAAGATCCGTGAGAA  R: CACTCCCCAATCCAAAGATG | 64 | Mehrtens et al. (2005) |
| *FLS* | At5g08640 | F: CCGTCGTCGATCTAAGCGAT  R: CGTCGGAATCCCGTGGT | 107 | Mehrtens et al. (2005) |
| *UDP-GST* | At4g14090 | F: GATCAGAGGAAGTGATCGAGGA  R: GCCAAAACAGCTGTCTGAGAA | 60 | Kleindt et al. (2010) |
| *UGT78D3* | At5g17040 | F: CTCCTCCGATATCCCCACAAA  R: TCAACACGAATCCCTCAGGAA | 71 | Sakakibara et al. (2008) |
| *GST* | At1g02920 | F: CTTCCGCAACCCTTTTGG  R: GGCTATGCCCGCAATGT | 154 | **In this study** |
| *UF3GT* | At5g54060 | F: CAACTGGTTTTCCGTTTCTGGTT  R: GCTTCCTCGACGGTTGATACAC | 64 | Solfanelli et al. (2006) |
| Acylation | At1g03495 | F: CTTCCCTGGAGCTGGAATCT  R: GCCGCTGGATTTGGTCA | 112 | **In this study** |
| Acylation | At1g03940 | F: CAGAGCCACTTTTACATTGAGC  R: TCATCCTTGTCTTCCTCGTTG |  | Luo et al., (2007) |
| Acylation | At3g29590 | F: AGCCACGCTCCTCCACTATC  R: ACGGCATCTTTGTCGTCAGG | 102 | Luo et al., (2007) |
| *SAT* | At2g23000 | F: CTGCTGCTATAGTCAAGTCTCTTCC  R: GAGAGAAGAACATCCAGGTCCTC |  | **In this study** |
| *RHM1* | At1g78570 | F: GGTGGGCGACACTTTGATG  R: CATATGGGTTTGACTTGGTTTTTCA | 78 | Sakakibara et al. (2008) |
| *RHM2* | At1g53500 | F: TCTACAATGTCGGCACAAAAAGA  R: TTCCCGAAAAGTTTGCAGATG | 73 | Sakakibara et al. (2008) |
| *RHM3* | At3g14790 | F: ATGCAGATGGTAATCAGACATTCAC  R: AGGATCTTTTGTCTCCGGAACA | 78 | Sakakibara et al. (2008) |
| *PAP1* | At1g56650 | F:AAATGGCACCAAGTTCCTGT  R:TCAGAGCTAAGTTTTCCTCTCTTGAT | 113 | **In this study** |
| *PAP2* | At1g66390 | F:GACTGCTGAAGAAGATAGTCTCTTG  R: GCCCAGCTCTCAAAGGAACTTGATG | 104 | Velten et al. (2010) |
| *MYB11* | At3g62610 | F: GGCGATTGTAACCCAAGCATT  R: TCACATGAGGACACGTGGACA | 116 | Gou et al.  (2011) |
| *MYB12* | At2g47460 | F: TGATGGGGAGTTGCATAACATA  R: AACGACTCCACCGATGGAC | 114 | **In this study** |
| *MYB111* | At5g49330 | F: AATAACAAGACCAAGAAGAAGAAGAA  R: AGAAACATTGTGAGGCCGTC | 92 | **In this study** |
| *MYB113* | At1g66370 | F: ATCTTGTTCTTCGCCTTCATAAA  R: GCATCGTTCATCGTGCTTCTTA | 134 | Gou et al.  (2011) |
| *MYB114* | At1g66380 | F: GTCTCTTGAGGCAGTGTATTGGT  R: TTTTCCTGCACCGATTTAGC | 87 | Qi et al.  (2011) |
| *GL3* | At1g17260 | F: AGTGTTTAGCCGTTCTCTTCTAGC  R: TGTCTTCCGTAATATGTTCTGTGG | 113 | Kleindt et al. (2010) |
| *EGL3* | At1g63650 | F:TTGGCACGACCGAACATA  R:TTGATAGTCTGATCTTGTCGATATTGT | 100 | Kleindt et al. (2010) |
| *TT8* | At4g09820 | F: TGAATCAACCCATACGTTAGACA  R: GGGGTGTGACATGAGAAGTGT | 102 | Kleindt et al. (2010) |
| *TTG1* | At5g24520 | F: TCCTCGAAGATTACAACAACCG  R: CGGGAGAGGCTTAACGGTCAT | 72 | **In this study** |
| *4CL1* | At1g51680 | F: TCAACCCGGTGAGATTTGTA  R: TCGTCATCGATCAATCCAAT | 132 | Bhargava et al. (2010) |
| *HCT* | At5g48930 | F: GCCTGCACCAAGTATGAAGA  R: GACAGTGTTCCCATCCTCCT | 136 | Bhargava et al. (2010) |
| *C3′H1* | At2g40890 | F: GTTGGACTTGACCGGATCTT  R: ATTAGAGGCGTTGGAGGATG | 104 | Bhargava et al. (2010) |
| *CCoAOMT* | At4g34050 | F: CTCAGGGAAGTGACAGCAAA  R: GTGGCGAGAAGAGAGTAGCC | 146 | Bhargava et al. (2010) |
| *CCR1* | At1g15950 | F: GTGCAAAGCAGATCTTCAGG  R: GCCGCAGCATTAATTACAAA | 153 | Bhargava et al. (2010) |
| *CCR2* | At1g80820 | F: GAACCCAACTGATCCCAAGA  R: ACAATGGCTTGAGTGTCACG | 303 | Vanholme et al.  (2010) |
| *F5H* | At4g36220 | F: CTTCAACGTAGCGGATTTCA  R: AGATCATTACGGGCCTTCAC | 87 | Bhargava et al. (2010) |
| *COMT1* | At5g54160 | F: TTCCATTGCTGCTCTTTGTC  R: CATGGTGATTGTGGAATGGT | 199 | Bhargava et al. (2010) |
| *CAD1* | At4g34930 | F: CCTCCATGAATGATCCGGATCTAA  GAA  R: CGAAATAAGGTAACTTGTCTGAGA  AGAAA | 153 | Sibout et al. (2005) |
| *CAD3* | At2g21890 | F: ATCGTTTCGGATATTGAGCTCATAAA  R: TCTCAGCTGACGACTCAGGGAGTAAA | 133 | Sibout et al. (2005) |
| *CAD4* | At3g19450 | F: TCTGGTGGAGGAGGCTGCAACA  R: AGCCAAAGCATTCGTGTTTGAACCA | 144 | Hossain et al., (2011) |
| *CAD5* | At4g34230 | F: TTGGCTGATTCGTTGGATTA  R: ATCACTTTCCTCCCAAGCAT | 164 | Bhargava et al. (2010) |
| *CAD7* | At4g37980 | F: CGATGAAGCCAACTCCTTAACTAGAAA  R: CAACTGATAAAGTACATGCAGTG  TGGTAATA | 141 | Sibout et al. (2005) |
| *CAD8* | At4g37990 | F: CATTGAAGCCTAATCCTAATTTATA  AGTTTTAA  R: ACTATTCATTTATTGGATTAAGCAT  ACCAAATTA | 144 | Sibout et al. (2005) |
| *LAC4* | At2g38080 | F: GGTGGATGGGTCGTCATGAGATTC  R: CGTGGCGTGATGTTGATATGTCGCCC | 213 | Sawa et al.  (2005) |
| *LAC17* | At5g60020 | F: GGCCATTTATCGGTTTGACAT  R: CAGAAAACAGAGCTGTGCAAC | 100 | Minic et al. (2009) |
| *SND1* | At1g32770 | F: CCCGAGTTCGCTTTCCAGGTG  R: CAGGACGAACCGGGCAACAGT | 141 | Hossain et al. (2011) |
| *MYB58* | At1g16490 | F: CCAGAGAACAGAGCTCTTCAAGAG  R: ATGTATGAGGAGCTCGTAACTCTC | 180 | Zhou et al. (2008) |
| *MYB63* | At1g79180 | F: GAACAGCTCAGGCTCAAGAGCAAC  R: ATGTATCATGAGCTCGTAGTTCTT | 178 | Zhou et al. (2008) |
| *EF-1α* | At5g60390 | F: CTGGAGGTTTTGAGGCTGGTAT  R: CCAAGGGTGAAAGCAAGAAGA | 72 | **In this study** |
| *Actin* | At3g18780 | F: AGCACTTGCACCAAGCAGCATG  R: ACGATTCCTGGACCTGCCTCATC | 156 | **In this study** |
