# Supplementary material for: Induced transcriptional profiling of phenylpropanoid pathway genes increased flavonoid and lignin content in Arabidopsis leaves in response to microbial products
Source: BMC Plant Biol. 2014 Apr 1;14:84. doi: 10.1186/1471-2229-14-84 (PMC4021374; doi:10.1186/1471-2229-14-84)

Additional file **1**

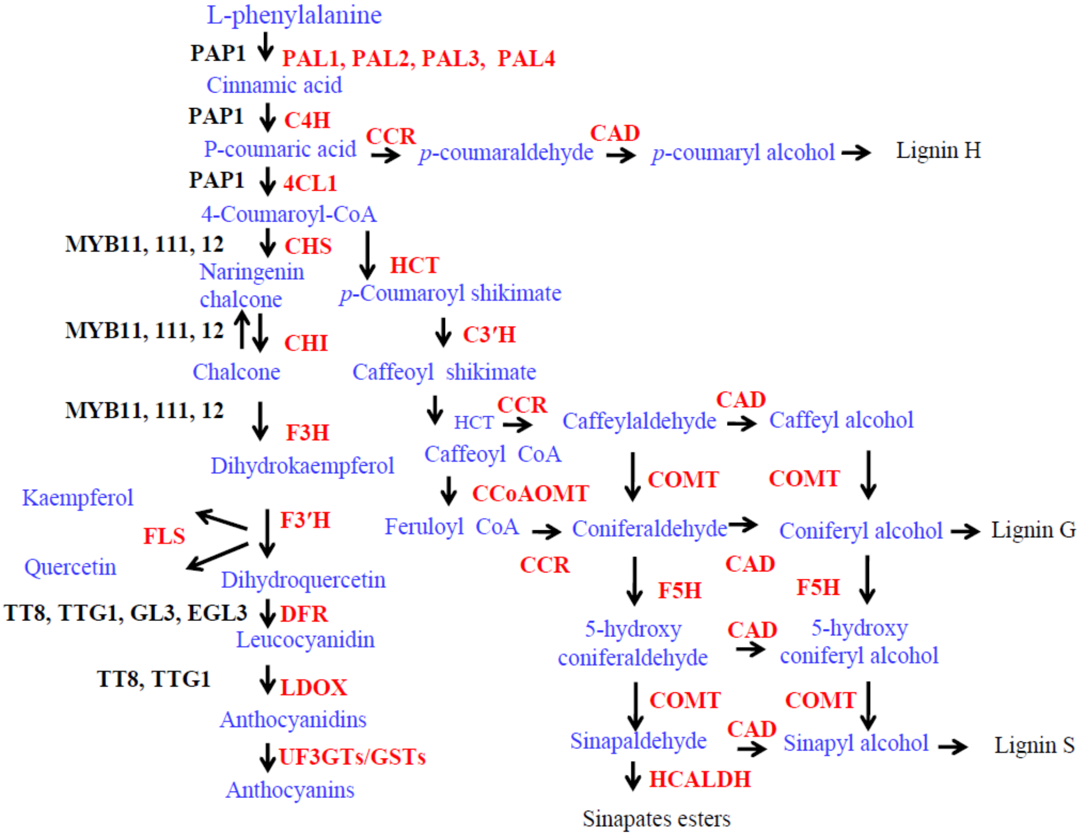

Supplement: Supplementary file 4 [file 1471-2229-14-84-S1.doc]
